# Macrophage membrane-functionalized nanotherapeutics for tumor targeted therapy

**DOI:** 10.7150/thno.108875

**Published:** 2025-03-31

**Authors:** Mubassir Khan, Razi Ullah, Guixue Wang, Maoquan Chu

**Affiliations:** 1 Key Laboratory of Biorheological Science and Technology of Ministry of Education, College of Bioengineering, Chongqing University, Chongqing, 400044, P.R. China.; 2 Key Laboratory of Biorheological Science and Technology of Ministry of Education, State and Local Joint Engineering Laboratory for Vascular Implants, Bioengineering College of Chongqing University, Jinfeng Laboratory, Chongqing, 400030, P.R. China.; 3 Research Center for Translational Medicine at Shanghai East Hospital, Frontier Science Center for Stem Cell Research, School of Life Sciences and Technology, Tongji University, Shanghai, 200092, P.R. China.

**Keywords:** macrophage membrane, nanotherapeutics, targeted delivery, immunological cell death, cancer therapy

## Abstract

Cancer is a multifaceted disease characterized by uncontrollable cell growth. To date, various therapies are employed including conventional chemotherapy, surgery, radiotherapy, and immunotherapies. However, these approaches still present significant limitations. Interestingly, macrophage membranes utilize their innate antigen recognition affinity to facilitate targeted localization to tumor sites with high specificity. As a result, they display distinct characteristics such as avoiding premature leakage, tumor targeting ability, immune evasion, immune system activation, tumor-infiltrating ability, improved cell endocytosis and release payload in tumor-microenvironment. In this paper, the recent advances in macrophage-membrane-encapsulated nanotherapeutics for targeted cancer therapy are presented. We begin by introducing macrophage membrane-encapsulated nanotherapeutics preparation and characterization, followed by cancer immunotherapy such as macrophage polarization, T-cell infiltration, macrophage membrane modification, immunization, and inducing immunological cell death. Lastly, a future perspective is proposed to highlight the limitations of macrophage membrane-encapsulated nanotherapeutics and the possible resolutions toward the clinical transformation of currently developed biomimetic chemotherapies. We believe this review may be beneficial for improving the deep research of macrophage membrane-encapsulated nanotherapeutics for targeted cancer therapy.

## Introduction

Cancer is a diverse disease characterized by uncontrolled proliferation of cells 1, and caused by a variety of factors, including environmental factors such as chemicals, radiation, pollution, and pathogens, as well as hereditary factors 2. Mutations in two types of genes, oncogenes and tumor suppressor genes, affect cell proliferation, development, apoptosis, and DNA repair, which can cause cancer. Tumor suppressor genes (TP53 and CDKN2A) control cell division and proliferation. Mutations in these genes lead to uncontrolled cell division and proliferation 3. Conventional chemotherapy is currently the first line of treatment or systemic-based therapy; nevertheless, chemotherapeutic drugs are associated with severe adverse consequences and relapses after the drugs are ceased 4, 5. Thus, alternative drugs with potential antitumor activity, low toxicity, and high therapeutic effectiveness are desirable for the successful treatment of cancer. In the past few years, immunotherapy has been an essential component of cancer research for fostering the introduction of de novo treatments rapidly converted into clinical outcomes 6. Immunotherapy employs the body's defensive mechanism to identify and kill cancer cells 7, and presents numerous significant advantages over conventional cancer therapies. Immunotherapy particularly targets tumor cells by augmenting the capability of the immune system to identify and eliminate them, while simultaneously offering long-term advantages via the development of immunological memory, consequently reducing the probability of recurrence. Immunotherapy is versatile, since it can be tailored to target tumor-specific antigens, which makes it effective against numerous cancers, even those resistant to traditional therapies. Moreover, its systemic strategy enables the targeting of metastatic cancer cells, often unreachable by localized treatments 8, 9. So far, various immune therapies have been introduced and further advanced in clinical trials. Yet, there are still several challenges that limit their clinical translation, including low efficacy, off-target side effects, and immune-related reactions 10. Cancer immunotherapy's effectiveness rate is highly influenced by a variety of variables, including individual immune response rate, immunosuppressive-tumor microenvironment (TME), and resistance development. However, by leveraging the potential of nanotechnology, such disincentive challenges might be addressed 11.

**Figure 1:**
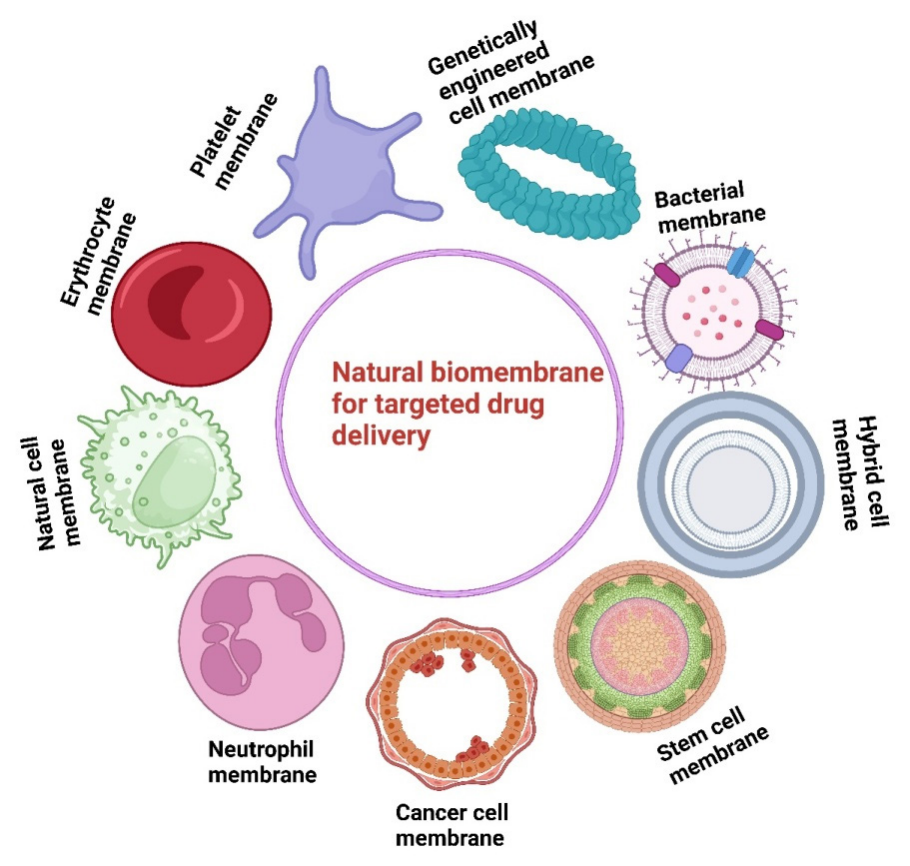
This figure highlights the development of natural biomembrane-based nanotherapeutics for tumor-targeted therapy during the past decade. It demonstrates the use of different cell membranes as drug carriers, emphasizing their potential to improve targeted drug delivery.

In this regard, numerous cell types have been considered as sources of membrane-cloaked nanotherapies as shown in **Figure 1** 12, including red blood cells (RBCs) 13, white blood cells (WBCs) 14, and stem cells 15. Bacteria-derived outer membrane vesicles 16, and cell-derived extracellular vesicles are also utilized for nanoparticle (NPs) modification to develop biomimetic drug-delivery systems 17. For example, cancer cell membranes derived from 4T1 cells and loaded with Bcl-2 inhibitor ABT-737 in poly-(lactic-*co*-glycolic-acid) (PLGA-NPs) induced higher levels of apoptosis in triple-negative breast cancer (TNBC) 18. 4T1-breast cancer cell membranes and paclitaxel-loaded polymeric-NPs (cell membrane coated-NPs), demonstrated superior interactions with its source tumor cells and effectively inhibited breast cancer growth and metastasis 19. Similar results have been obtained from the biomimetic NPs (indocyanine green [ICG] loaded and MCF-7 cell membrane coated NPs) that showed unique adhesion to MCF-7 cells and exhibited excellent fluorescence intensity 20. However, there are several limitations to cancer cell membrane-coated targeted-drug delivery systems. These include, differences in membrane composition from different cancer cells which may lead to inconsistent therapeutic effects and the presence of tumor-derived proteins which some host immune cells still recognize and clear the nanoparticles. Although these are suitable biocompatibility of cell membrane-coated drug delivery systems, their physiological behavior during long-term circulation has not been described, hence, the biological safety of biomimetic nanodrug delivery requires additional exploration 21.

The encapsulation of leukocyte-membrane with nano-porous-silica-NPs yielding leukolike vectors characteristics include membrane-coating, and multiple receptors required for crosstalk with the endothelium, enabling cellular aggregation within TME while minimizing the vascular barrier. However, some tumors develop immunosuppressive environments, which may neutralize leukocyte-based targeting strategies 22. Excessive shedding of leukocyte membrane vesicles in cancer promotes immune evasion, tumor growth, and metastasis by carrying immunosuppressive signals and adhesion molecules 23. Furthermore, the direct utilization of leukocytes such as neutrophils as drug carriers has limitations because of its short half-life 24. Platelet membrane (PM) coated nano-vehicles for the simultaneous administration of extracellular-Tumor necrosis factor (TNF)-related apoptosis-inducing ligand and intracellular-Dox (TRAIL-Dox-PM-nanovesicles system) face several limitations 25. These include potential activation during drug acquisition, separation, processing, and storage, as well as alteration of the drug's chemical structure or premature release, which may compromise therapeutic efficacy and even inducing thrombosis 26. Platelet-membrane drug delivery approaches are unable to entirely bypass the immune system, accumulating in the liver and kidney 27, 28. The employing of mammalian natural killer (NK) cells to wrap synthetic drug delivery NPs in biomimetic design has drawn significant interest in the delivery of chemotherapeutic drugs to specific tumor sites while integrating cell biological complexity. NK membrane camouflage is employed to cover carboxylate-terminated poly (latic-co-glycolic acid) (PLGA) biomimetic nano-constructs 29. Gadolinium contrast materials and near-infrared (NIR) dyes later were added to the nanoconstructs, and their imaging features were assessed employing magnetic resonance imaging and NIR fluorescence. Confocal imaging and cell sorting studies demonstrated NK membrane-camouflaged system interacted differently with MCF-7 cells compared to plain polymeric NPs. The current adoptive cell therapies have substantial disadvantages in terms of tumor invasion, which restricts their efficacy 30.

Macrophages, as diverse and versatile immune cells, are vital to several biological processes, including the maintenance of tissue homeostasis, regulation of cancer growth, and the defense against diseases. Their phenotype and functioning are tightly regulated by the surrounding milieu, and macrophages exhibit both antitumor and tumor-promoting activities in the context of cancer 31, 32. Macrophages shift among their two primary phenotypes, M1 and M2, in response to cytokine or pathogenic signals. These two macrophage morphologies have opposite functions: M1-macrophages are pro-inflammatory, immunogenic, and anticancer, whereas M2-macrophages are anti-inflammatory, tolerogenic, and pro-tumor 33. Tumor-associated macrophages (TAMs), which are macrophages that exist in TME, play a pivotal role in cancer progression and metastasis. Most TAMs display M2-like phenotype in response to local cytokine mediators, promoting tumor angiogenesis, immune surveillance evasion, and matrix remodeling enabling tumor development. Therefore, poor clinical outcomes are related to significant TAM infiltration in tumor tissue 34. TAM-related anti-cancer treatment includes TAM generation and viability suppression, as well as TAM phenotypic modification 35, 36. The TAM phenotypic shift is considered to be less hostile and more successful than the others since TAMs (M2-type) polarized into M1-type macrophages by a variety of procedures 37,38. Furthermore, this transition inhibits tumor development and destroys tumor-supporting TME 39. As a result, TAM reprogramming to the M1 type has become popular as a cancer therapy method 40.

The integration of the macrophage membrane (MΦM) offers a multifunctional approach to tumor-targeted therapy. The coating of MΦM prevents uptake by the immune system and premature leakage, as well as improves the interaction between NPs and cancer cells via membrane proteins, extending the circulation time, reducing nanomedicine toxicity and enhancing targeted delivery 41, 42. Taking advantage of the biocompatibility and versatility of MΦM, these nanoparticles retain inherent proteins and receptors, enabling targeted interactions with tumor cells and immune modulation, making them excellent candidates for tumor-targeting 43, 44. NPs camouflaged with MΦM not only improve cell-cell adhesion for tumor targeting, but they can also evade the immune system.

Tumor-associated macrophage membranes (TAMMs) are employed to coat photosensitizer-loaded up conversion NPs (UCNPs) to attach colony-stimulating factor-1 (CSF-1), typically targeting native TAMs. As a result, blocking the CSF1/CSF1R axis changeds macrophage activation from tumor-promoting M2 to tumor-killing M1 phenotype 45. Macrophages are beneficial for drug administration due to their vulnerability to hypoxia, active migration, and tendency to infiltrate tumors 46. SIRPα-overexpressed cell membranes with M1 polarization were developed to coat microwave-responsive Prussian blue (PB) nanoparticles (SIRPα-M@nanoPB). This novel design enables highly targeted delivery to osteosarcoma cells and has synergistic effects in the therapy of osteosarcoma via numerous mechanisms such as microwave hyperthermia, microwave dynamic effect, and immunological modulation. The modified MΦM targets tumor cells and blocks the CD47-SIRPα checkpoint, overcoming immune suppression. The microwave-responsive nature of the modified nanoPB allows for precise and controlled heating of the tumor, resulting in localized hyperthermia efficiently killing osteosarcoma cells 47.

Particularly in all types of cancer, the infiltration of TAMs is substantial and correlated with increased tumor aggressiveness, metastasis, and lower overall survival rates 48. Targeting TAMS via MΦM-encapsulated nanotherapeutics is a highly suitable strategy in cancer immunotherapy and harnesses' the natural properties of macrophages to achieve targeted, efficient, and biocompatible delivery of therapeutic agents directly to TME.

Building on the unique properties of MΦM, which include avoiding premature leakage, tumor targeting ability, escape from the immune system, immune system activation, tumor-infiltrating ability, and improved cell endocytosis. This review highlighted MΦM-based nanotherapeutics for immunotherapy of cancer-targeted therapy, including influencing T-cell activity, macrophage polarization, and anti-HER2 targeting and immunogenicity. We further explored various TME-based approaches for targeted cancer treatment such as MΦM-conjugated biomolecules, inducing immunological cell death, gas therapy, and the theranostics approach. Furthermore, the potential obstacles and prospects involved in MΦM base-targeted cancer therapy are discussed.

## Preparation and characterization of macrophage membrane based nanotherapeutics

**Figure 3:**
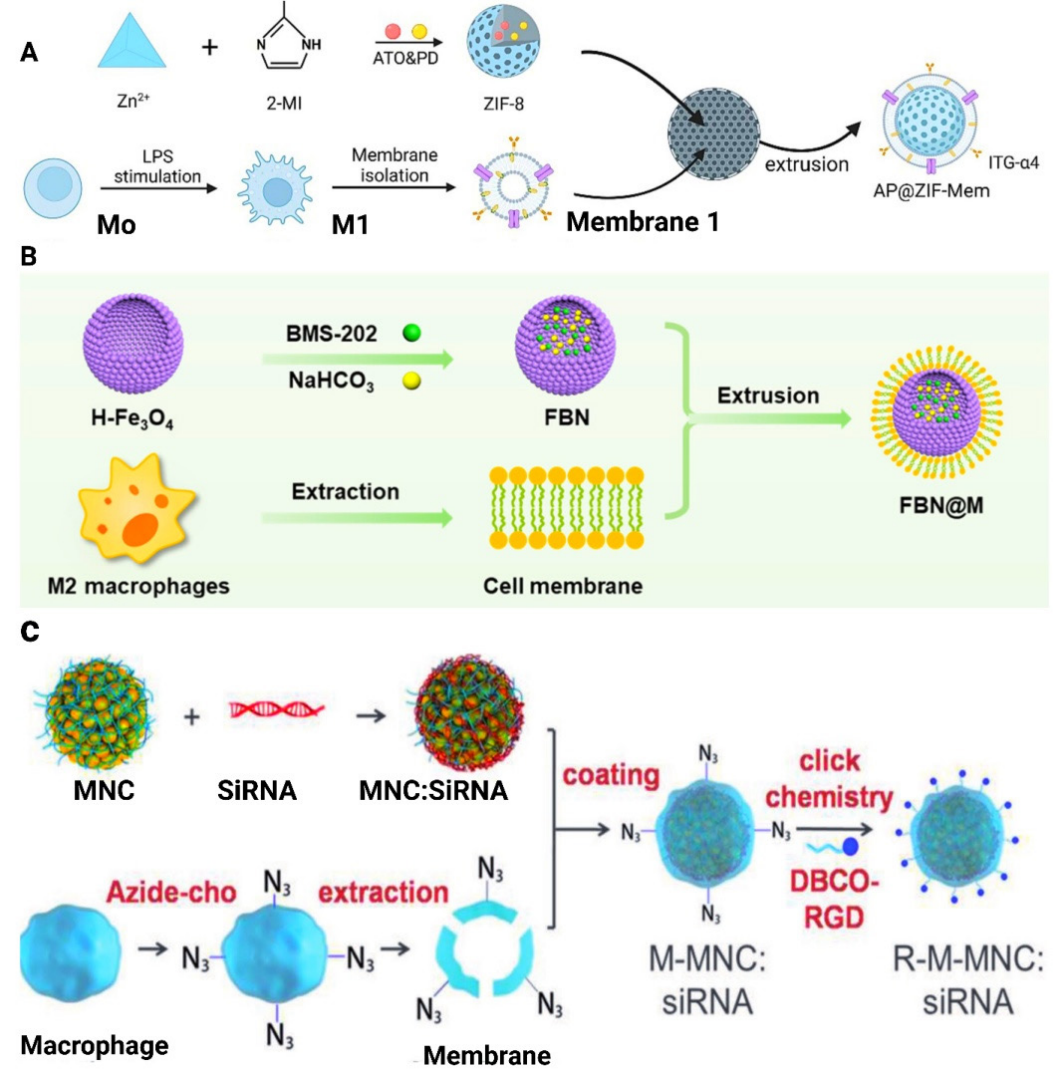
Preparation methods for MΦM. (A) Schematic diagram of the preparation of atorvastatin and polydatin co-loaded MΦM-coated metal-organic framework NPs (AP@ZIF-Mem). Reproduces with permission 51. Copyright 2024, Elsevier. (B) Extrusion method to create FBN@M. Reproduce with permission 52. Copyright, 2024 American Chemical Society. (C) Synthesis strategy through the combination of MNC synthesis, an engineered membrane with an azide group, electrostatic assembly, and click reaction. Reproduced with permission 53. Copyright 2018, Wiley Online Library.

Cell membrane-camouflaged NPs are mostly composed of thin layers of cell membranes, loaded with therapeutic NPs. This forms a "shell and core" structure with NPs serving as the core and the membrane as the outermost shell. The preparation of MΦM-coated NPs mainly involves the followng steps: (1) macrophage cell extraction and/or culture, (2) extraction and isolation of the cell membrane, and (3) formation of membrane-wrapped NPs 49, 50. A study by Qing et al. for example, isolated MΦM from the cultured RAW 264.7 cells and were subjected to ultrasonic disruption with a cell disruptor of around 20% power to ensure entire cell destruction. The supernatant was collected and centrifuged at 14,000 rpm at 4°C for 30 minutes in order to get the cell membrane. The MΦM was then added to AP@ZIF-8 and sonicated for 2 minutes. The solution was then extruded over ten times via a 200 nm polycarbonate porous membrane to yield AP@ZIF-Mem, which is subsequently dissolved in water for future applications as shown in Figure 3A 51. The one-step hydrothermal approach is employed to develop hollow Fe_3_O_4_-NPs, incorporating citrate as a reductant. BMS-202 (a small-molecule PD-1/PD-L1 inhibitor) and NaHCO_3_ were incubated with hollow Fe_3_O_4_-NPs to produce FBN-NPs (Figure 3B). FBN-NPs were combined with MΦM and repeatedly extruded onto a porous polycarbonate membrane having a pore diameter of 220 nm to create FBN@M 52. Synthesis of RGD-Membrane MNC:siRNA (R-M-MNC:siRNA) as shown in Figure 3C develops via extraction, coating, and click chemistry methods. Simply siRNA is adsorbed onto magnetic nanoclusters (MNCs) to form MNC:siRNA complexes. To achieve this, MNCs dissolved in RNase-free dH2O are incubated with siRNA and then purified using magnetic separation to discard free siRNA. MNCs:siRNA was then added to the previously prepared PEI solution (6% w/v). PEI molecules bind to MNC:siRNA by electrostatic interaction, converting the negatively charged surface to a positive one. The PEI-modified MNCs:siRNA complexes are incorporated with MΦM, washed, and magnetically separated. Before the development of R-M-MNC:siRNA, DBCO-Mal and Arg-Gly-Asp (RGD) peptides were dispersed in phosphate-buffered saline (PBS) and heated to 25°C. Free DBCO-Mal and RGD are eliminated by dialysis. The resulting DBCO-Mal-RGD was then incubated with M-MNC:siRNA at room temperature before being collected by magnetic separation 53. A multifunctional cell-mimicking nanostructure was developed *via* hierarchical self-assembly to enhance the therapeutic efficacy against breast cancer. PH-sensitive Dox-MPK prodrugs were synthesized and inserted into the double-stranded structure of the DNA tetrahedron dendrimer. Then, the DNA skeleton is successively coated with liposomes and MΦM to form Dox-MPK@MDL and equipped with targeting protein and bio-interfacing properties of macrophage cells. Dox-MPK@MDL is anticipated to mimic the source cells and target the metastatic sites in breast cancer and trigger Dox release from the prodrug for suppressing metastasis 54. MΦM-encapsulated pH-responsive zeolitic-imidazolate framework-8 (ZIF-8)-loaded naringenin (Ng-NPs) (MM-ZIF-8@Ng) were developed for tumor-targeted drug delivery to treat breast cancer. The MΦM encapsulation allows the nanomedicine to evade recognition by the immune system and surface proteins for enrichment at the tumor site to enhance targeting. The drug-loaded biomimetic NPs exhibited the highest release rate in the TME. This increased uptake is due to the presence of α-4 integrin in the membranes, which recognizes VCAM-1 receptor on the cancer cells, increasing the accumulation of the NPs at the tumor site 55.

**Figure 4:**
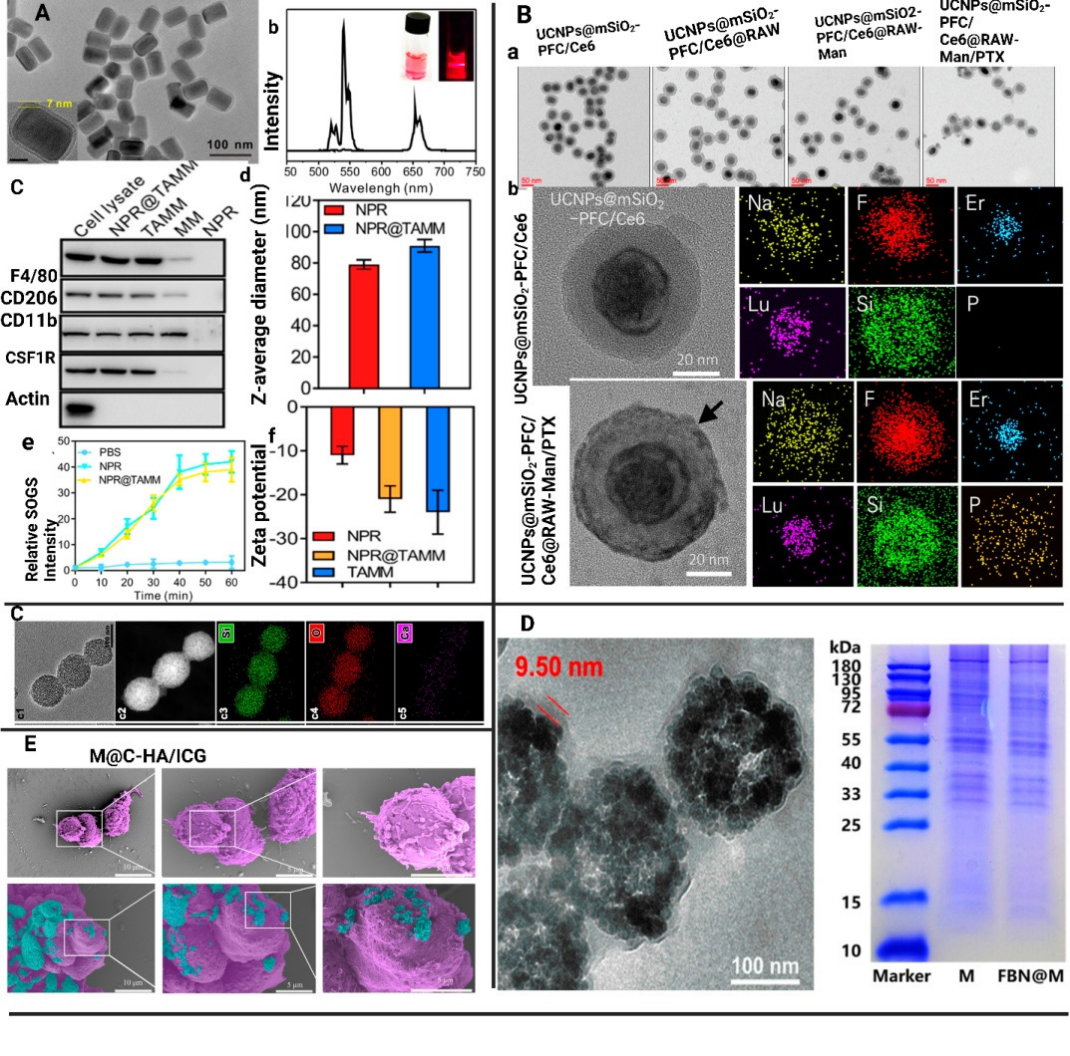
MΦM-coated NP characterization. (A) NPR@TAMMs images (a) TEM analysis of NPR@TAMMs. (b) The up-conversion emission spectrum of UCNPs and NPR@TAMMs. (c) Western blotting for quantification of different cell membrane markers for NPR, cell lysate, TAMMs, and NPR@TAMMs. (d) Hydrodynamic size of NPR@TAMMs. (e) Generation of singlet O_2_ by NPR@TAMMs based on the fluorescence intensity. (f) Zeta-potential of NPR@TAMMs. Reproduced with permission 45. Copyright 2021, American Chemical Society. (B) (a) TEM analysis of UCNPs@mSiO_2_-PFC/Ce6, UCNPs@mSiO_2_-PFC/Ce6@RAW, UCNPs@mSiO_2_-PFC/Ce6@RAW-Man, and UCNPs@mSiO_2_-PFC/Ce6@RAW-Man/PTX. (b) Magnified TEM images of UCNPs@mSiO_2_-PFC/Ce6 and UCNPs@mSiO_2_-PFC/Ce6@RAW-Man/PTX and their element mappings for Na, F, Er, Lu, Si, and P. Reproduced with permission 58. Copyright 2023, Elsevier. (C) RAW M@MBG (c1-6). Reproduced with permission 59. Copyright 2023, Elsevier (D) TEM image of FBN@M and SDS-PAGE protein analysis of MΦM on FBN@M. Reproduced with permission 52. Copyright 2024, American Chemical Society. **(E)** M@C-HA/ICG SEM analysis of M, and M@C-HA/ICG. Reproduced with permission 60. Copyright 2022, Elsevier.

The MΦM-coated NPs underwent additional investigation to validate their effective coating. Physicochemical and biological properties, such as transmission electronic microscopy (TEM), scanning electronic microscopy (SEM) analysis, size distribution, zeta potential, stability, and membrane protein activities, as well as the functional performance of MΦM-coated NPs were systematically evaluated to determine their efficacy in targeted delivery and immune modulation. For example, Geng et al. developed MΦM-coated HMSNs (MA@RT-HMSNs) via a three-step process. Initially, TC-DAPK6 a potent ATP-competitive, and highly selective DAPK inhibitor, and fluorochrome rhodamine B (RhB) was loaded into hollow mesoporous silica nanoparticles (HMSNs) to obtain RT-HMSNs. Subsequently, MΦM were wrapped onto the surface of RT-HMSNs via extrusion. The resulting MA@RT-HMSNs displayed uniform spherical morphologies with a zeta potential of -19.3 mV, closely matching the MΦM vesicle potential of -16.5 mV, confirming successful membrane coating. Western blot analysis indicates strong expression of MΦM markers CD11b and F4/80 in macrophages, MAs, and MA@RT-HMSNs, demonstrating the retention of MΦM functionalities. The average diameter of MA@RT-HMSNs is approximately 91 nm, and their hydrodynamic diameter increased from 122 nm to 190 nm, with a polydispersity index (PDI) of 0.457. Thermogravimetric analysis (TGA) revealed an initial weight loss at approximately 250°C, highlighting the suitable thermal stability of the HMSN substrate. Additionally, most surficial cell membrane proteins were retained, and the extrusion process did not alter the protein profile, ensuring the structural and functional integrity of the nanoplatforms 56. For another example, BTPT-ASO_VEGF_-NPs are electrostatically co-assembled topotecan-hydrochloride (TPT) and vascular-endothelial growth factor antisense-oligonucleotide (ASO_VEGF_) at 10:1 molar ratio in sterile deionized water, followed by mechanically co-extrusion with MΦM resulting in TPT-ASO_VEGF_@MM-NPs. TEM images revealed spherical TPT-ASO_VEGF_-NPs with an average diameter of 142.9 ± 17.1 nm and a small gray coating on their surfaces, confirming MΦM coating and preserving their spherical shape. The average diameter of TPT-ASO_VEGF_@MM-NPs was 174.9 ± 9.8 nm, 32 nm greater than TPT-ASO_VEGF_-NPs and 15.1 nm MΦM layer thickness. Additionally, TPT-ASO_VEGF_-NPs and TPT-ASO_VEGF_@MM-NPs in water have diameters of 201.8 ± 34.5 and 233.2 ± 25.8 nm, respectively, with polydispersity index (PDI) values of 0.278 ± 0.09 and 0.197 ± 0.13, as determined by dynamic light scattering 57. Figure 4 illustrates the characterization of MΦM-functionalized nanotherapeutics developed for targeted cancer treatment.

## Macrophage membrane base nanotherapeutics for cancer immunotherapy

MΦM-based nanotherapeutics are a promising approach in cancer immunotherapy, which typically involves NPs coated with MΦM influencing the TME and boosting anti-tumor immune responses, potentially reversing the tumor's immunosuppressive nature 61. Here we discuss different strategies and methodologies of MΦM-coated nanotherapeutics for cancer immunotherapy.

### Targeting tumors and polarizing M2 tumor-associated macrophages to M1 phenotype

Macrophage polarization is a mechanism by which macrophages adopt multiple active states in response to various signals from their environment, critically regulating immune responses, cancer development, and therapy 62. MΦM derived from the M1 phenotype presents improved targeting and immunological activation, promoting effective tumor eradication and immune system activation and enhancing the delivery of drugs while reducing immune suppression. MΦM deriving from the M1 phenotype provides a physiologically relevant platform for targeted cancer immunotherapy by preserving immune-stimulatory characteristics, augmenting tumor-specific cytotoxicity, and facilitating pro-inflammatory cytokine secretion. For example, camouflaged black phosphorus (BP) with M1-MΦM and loaded with Histone deacetylase inhibitors (HDACi) suberoylanilide hydroxamic acid (SAHA) has been explored for targeted lung cancer therapy. The camouflage by M1-MΦM increased the efficiency and selectivity of cellular uptake toward lung cancer 63. Yao et al. synthesized atorvastatin and polydatin-loaded MΦM-coated metal-organic framework NPs (AP@ZIF-Mem) that accumulate at the tumor site via macrophage biomimetic activity. After tumor cell endocytosis, the NPs released atorvastatin and polydatin. Atorvastatin blocks MCT4, which is a lactic acid transporter involved in glycolysis, hence increasing tumor acidity and interrupting energy supply. Whereas, polydatin disrupts intracellular redox state, influencing the tumor's immunosuppressive microenvironment 51. Multilayer sono-responsive M1/IR780@PLGA-NPs with PLGA acting as a shell are loaded with IR780 as a sensitizer and encapsulated inside M1 macrophage-derived nano-vesicles to make sure IR780 loading in the nanocarriers and avoid premature leakage before reaching the tumor region. The modified M1-derived nano-vesicles quickly target tumor tissues and repolarize M2 in the TME into M1, stimulating anticancer immunological responses 64. Mag+NIR+DOX@MPN upregulates pro-inflammatory M1 biomarkers (iNOS and TNF-α) while downregulating anti-inflammatory genes (Arg-1 and IL-10) and Arg-1 protein expression level in mice 65. M1/PLGA@IR780 and M1/PLGA@IR780/CAT exhibited high sonosensitivity and boosted pro-inflammatory cytokines (TNF-a and IL-6) secretion, while anti-inflammatory cytokine IL-10 dropped by 22.9% and immunosuppressive cytokine TGF-β by 46.7%, indicating effective tumor immunity stimulation 66. The nanocarrier (PLGA-ION-R837@M (PIR@M)) is developed by loading PLGAION-R837 (PIR)-NPs with a diameter of 13 nm oleic acid modified Fe_3_O_4_-NPs (ION) and R837. Subsequently, these NPs are coated with M1-macrophage membranes from lipopolysaccharide-treated macrophages. PIR@M nanocarriers ingest Fe_3_O_4_-NPs, which stimulate IRF5 signaling pathways, whereas R837 stimulates the NF-κB signaling pathway, leading to increased polarization 67. Integrating advanced nanotechnology with immunomodulatory nanoplatforms with enhanced stability, specificity, and controlled drug release to effectively reprogram M2 macrophages into the M1 phenotype.

**Figure 6:**
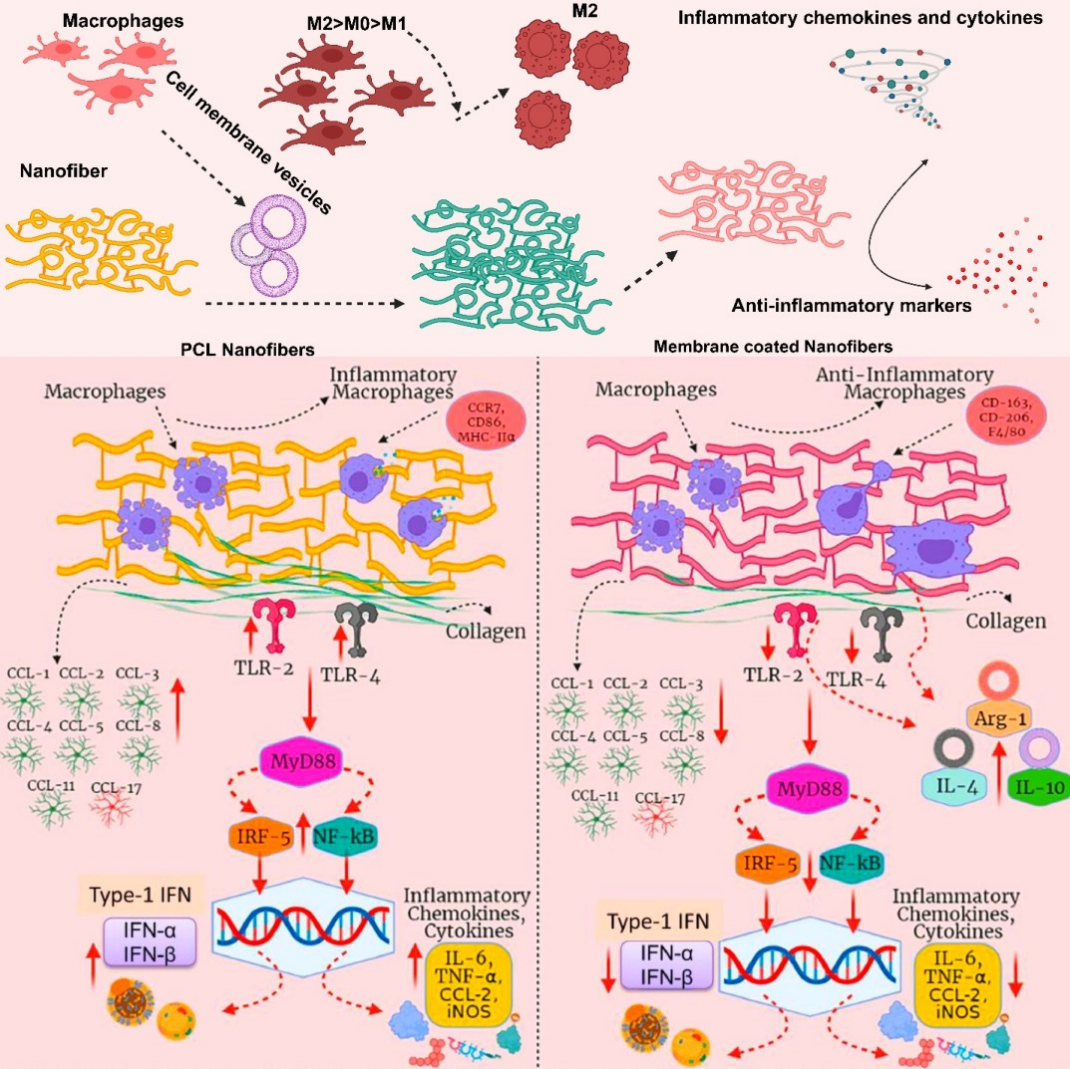
Immunomodulatory effects of MΦM-functionalized PCL nanofibers. The PCL nanofibers are wrapped with MΦM from different macrophages M0, M1, and M2 phenotypes. Various types of influences of the two types of nanofibers are presented for a direct comparison. Reproduced with permission 68. Copyright 2022, Elsevier.

MΦM from various phenotypes (M0, M1, and M2) were coated onto poly-ε-caprolactone (PCL) (Figure 6) nanofibers in order to gain external surface proteins and mimic natural membrane activities. M2-PCL nanofibers reduce inflammatory markers including TNF-α and IL-1β, while boosting anti-inflammatory markers like IL-10, Arg-1, and TGF-β 68. Uncoated selenylated Poria cocos polysaccharide lipid nanoparticles (Se-PP) develop by thin-film hydration from selenylated Poria cocos polysaccharide nanoparticles (Se-P) with liposome. These NPs are than coated with MΦM to make MΦM-Se-PP alleviate inflammation by suppressing the expression and secretion of pro-inflammatory factors in macrophages while simultaneously reversing the autophagy pathway, thereby restoring cellular homeostasis and immune balance 69.

Lin Hou et al. developed a hollow mesoporous Prussian blue (HMPB) nanoplatform (Man-HMPB) encapsulated in hydroxychloroquine (HCQ) (Man-HMPB/HCQ) for TAM targeting and polarization. Man-HMPB/HCQ greatly increases TAM cellular internalization and shifts M2 to M1 phenotypes with hybrid MΦ and thylakoid (TK) membrane-camouflaged Man-HMPB/HCQ, known as TK-M@Man-HMPB/HCQ. This exhibits MΦM trafficking function and reduces RES intake, alleviates hypoxia, and triggers membrane rupturing in TME 70. Zhang's group developed 4T1 and RAW264.7 cell membranes, resulting in a 4T1-RAW264.7 composite biomimetic coating effectively applied to hollowed iron oxide NPs loaded with R837 and ICG, designated RIFe@TRM. The RAW264.7 membrane greatly improves immunological capability, whereas the 4T1 cell membrane improves its specific target ability for breast cancer. Iron oxide NPs act as carriers for R837 and ICG, and catalysis for modifying the TME 61. MΦM-coated NPs loaded with the kinase inhibitor TGF-βR1, SD-208 (Mϕ-SD-NP), shifted cold tumors into hot tumors by specifically targeting tumors and blocking M2-type macrophage differentiation and development 71. Development of cancer cell macrophage hybrid-membrane-coated drug-delivery nanosystem for pancreatic cancer therapy. The siIRAK4/Er@GEM-SS-PC-M drug was produced by combining gemcitabine (GEM) with a cationic polymer gene vector (PC) via a GSH-responsive linker. Erlotinib (Er) is then encapsulated via host-guest molecular interaction and loaded with siRNA to generate a cell membrane-coated nano-drug (siIRAK4/Er@GEM-SS-PC-M) that downregulates p65, which is necessary for macrophage polarization 72.

Biomimetic multifaceted nano-system designed to change the post-photothermal therapy (PTT) inflammatory milieu, with the ultimate objective of restoring immunosuppression and eliminating tumors following PTT. Initially, poly-dopamine NPs (PDANPs) were generated by oxidative polymerization of dopamine monomers. The TAM repolarizing agent TMP195 is attached to PDANPs, resulting in TMP195 containing PDANPs (P/T NPs). Subsequently, P/T-NPs were coated with MΦM (P/T@MM-NPs). The surface decorating with MΦM enhanced P/T@MM-NPs' capability for targeting the post-PTT inflammatory milieu and enhanced immune modulating capabilities considerably boosting the level of M1-like TAMs, eventually leading to 60% tumor elimination 73. Curcumin is a natural chemical compound that exhibits anti-cancer potential, but its limited solubility and bioavailability restrict its therapeutic use. To address these issues, Xi Liu et al. developed Cur-PDA@CM, a unique NPs system using curcumin self-assembly, polydopamine modification, and MΦM coating. The results showed that MΦM enhanced the NPs' potential to target tumor tissues, inhibiting tumor development and inducing apoptosis by curcumin release and polarizing TAMs towards the M1 phenotype 74. Based on the chelating metal ion properties of PDA, arsenic was immobilized on the organic carrier, and the M1-like MΦM-camouflaged manganese-arsenic complex mesoporous polydopamine (MnAsOx@MP@M) nanoplatform regulated the TAMs immune promotion 75. Hollow Fe_3_O_4_-NPs develop by loading BMS-202, a small molecule PD-1/PD-L1 inhibitor and pH-sensitive sodium bicarbonate (NaHCO_3_) into their inner cavity and then encapsulating it with MΦM to form biomimetic nano-systems (FBN@M) that target the tumor site based on MΦM innate inflammation directed chemotaxis, transform TME from immunosuppression to immunostimulant, and effectively block the PD-1/PD-L1 pathway to strengthen cancer immunotherapy 52. Mannose decorated/MΦM coated-silica layered Na-ErF4@Na-LuF4 up-converting nanoparticles (UC-NPs) co-doped with perfluorocarbon (PFC)/chlorin e6 (Ce6) and loaded with PTX (UC-NP@mSiO_2_-PFC/Ce6@RAW-Man/PTX) possess excellent targeting to M2-type (TAMs) and trigger polarization to M1 type macrophages, which subsequently release pro-inflammatory cytokines and suppress breast cancer 58. M@ZIF-8@miR nano-therapeutic system utilizes nanoscale zeolitic-imidazolate framework (ZIF) as a carrier for miRNA delivery, enclosed inside M2-membranes augmented its anti-inflammatory effects. M@ZIF-8@miR substantially reduced pro-inflammatory cytokines, controlled inflammation and enhanced anti-inflammatory M2 polarization of macrophages 76.

### T-cell infiltrations

**Figure 7:**
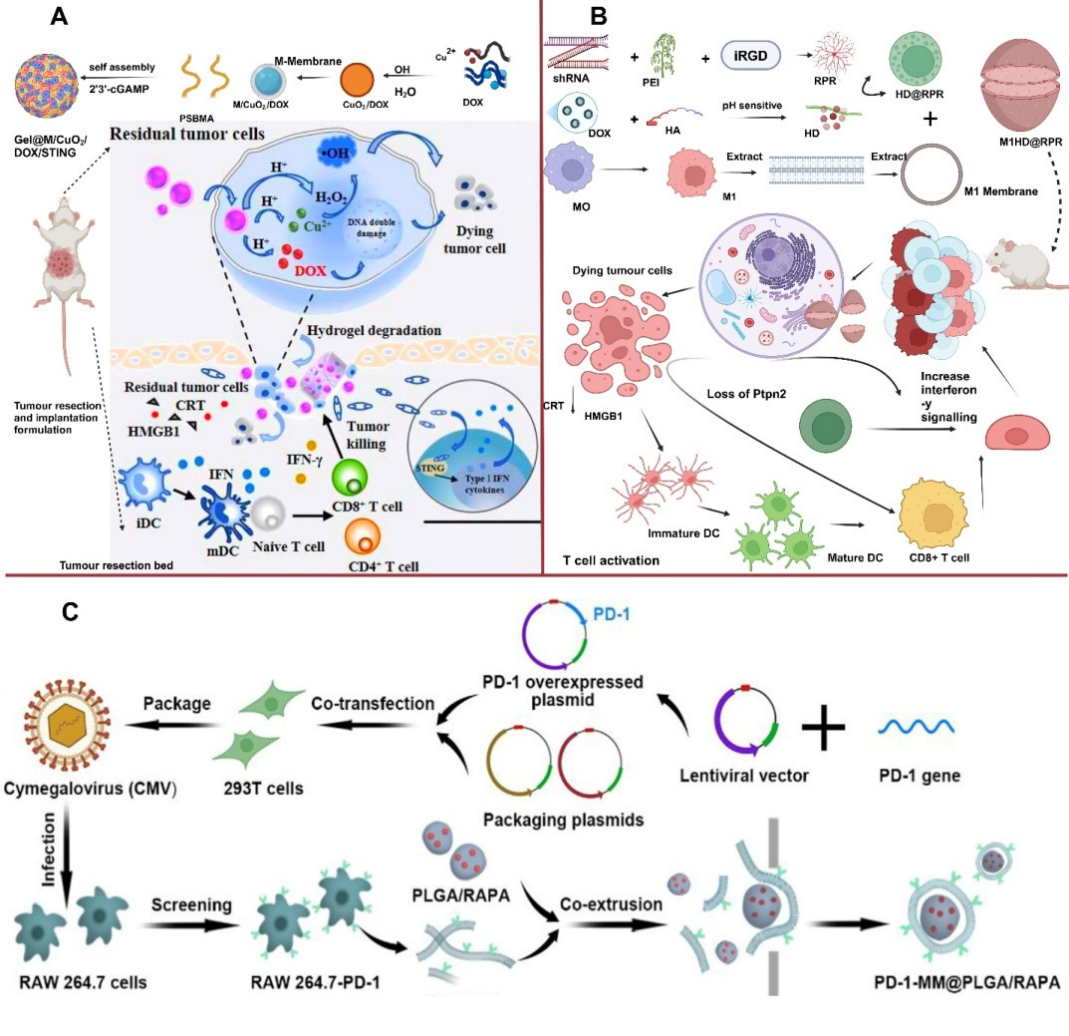
T-Cell Infiltrations (A) Schematic Illustration of the Drug-Loaded Hydrogel (Gel@M/CuO_2_/DOX/STING) for Preventing Postoperative Tumor Recurrence and Metastasis. Reproduced with permission 81. Copyright 2023, American Chemical Society. (B) M1HD@RPR preparations and its targeted mechanism in combined cancer therapy. Reproduced with permission 82, copyright 2022, science. (C) Schematic diagram of the synthesis of RAW 264.7-PD-1 and PD-1-MM@PLGA/RAPA and engineered MΦM-coated-NPs with enhanced PD-1 expression. Reproduced with permission 77. Copyright 2022, American Chemical Society.

T-cell infiltration refers to the presence and migration of T-cells into TME. Overcoming T-cell exhaustion is a primary goal of cancer immunotherapy, For example, Tieying Yin's research team designed MΦM-coated nanoplatform and confirmed programmed cell death-1 (PD-1) (PD-1-MM@PLGA/RAPA) (Figure 7C), which accumulates at the tumor site, boosting immune response via increasing CD8+ cytotoxic T-lymphocyte (CTL) infiltration 77. Tingting et al. synthesized TAMs membrane-camouflaged pH-responsive DOX-loaded hyaluronic-acid (HA)-g-poly (histidine) polymeric-micelles (DHP@M2) that accumulates within tumor sites via TAM membrane-mediated immune camouflage. In acidic TME, the particle size of DHP increased due to decreased hydrophobic interaction with the inner core, resulting in the "membrane escape effect" exposing the inner HA residue. DHP@M2 is capable of dual-targeting CD44/VCAM-1, allowing for intracellular DOX accumulation. Meanwhile, TAM membranes absorb colony-stimulating factor 1 (CSF1) via increased expression of its receptor (CSF1R) on TAM membranes, reducing TAMs in tumor tissues and relieving TIM. This approach effectively induced CTL infiltration for an anti-tumor immune response and inhibited tumor growth in 4T1 tumor-bearing mice 78. MΦM-coated NPs loaded with SD-208, a TGF-βR1 kinase inhibitor (Mϕ-SDNPs) increased the proportion of CTLs in tumor tissue, leading to improved immune response and a strong anticancer effect when combined with anti-PD-1 antibodies 71. LCL161-loaded MΦM-coated-nanoparticles (LMN) for the treatment of MHC-I deficient triple-negative breast cancer. SIRPα on the surface of MΦM helps LMNs identify CD47 expressing cancerous cells for targeted delivery of LCL161, boosting intratumoral concentration of CTL lymphotoxin by 4.6-fold and inhibiting growth and development of MHC-I deficient TNBC tumors, along with combined therapy of anti-PDL1 antibody and albumin-bound paclitaxel. The MΦM-decorated NPs provide a broad platform for boosting macrophage-mediated anti-tumor immunity, thus allowing for successful immunotherapy of MHC-I-deficient tumors 79. Core-shell anti-phagocytosis-blocking repolarization-resistant membrane-fusogenic liposome and M1- MΦM (ARMFUL/M1) MΦM presents CD47, which enhances macrophage phagocytosis towards the tumor. Blocking CD47 changes the TME, stimulates T-cell cytotoxicity and generates immunological memory to simultaneously limit tumor development following adoptive transfer 80.

Fang et al. loaded DOX into copper peroxide NPs (CuO_2_/DOX), and subsequently coated them with MΦM to develop tumor-targeting NPs (M/CuO_2_/DOX). M/CuO_2_/DOX and STING agonist 2′, 3′-cGAMP were co-loaded into PSBMA hydrogel (Gel@M/CuO_2_/DOX/STING) (Figure 7A). Body fluid in TME influences ion interaction of the hydrogel skeleton, causing the hydrogel to disintegrate and release small NPs (M/CuO_2_/DOX) and 2′,3′-cGAMP followed by tumor cell ingestion. The NPs in the acidic environment cause M/CuO_2_/DOX to burst and release DOX and Fenton catalyst (Cu^2+^ and H_2_O_2_) inducing DNA damage, which triggers the STING pathway and promotes type-I IFN generation. These inflammatory mediators boost DC maturation and increase tumor-specific T-cell infiltration, therefore altering the immunosuppressive TME. In the present study, CDT is paired with STING pathway activation in a drug-loaded hydrogel-carrying system to effectively destroy tumor cells and restructure immunosuppressive TME to trigger anti-tumor immunity 81. Encapsulating pH-sensitive DOX and nuclei targeting shRNA-Ptpn2 with M1-MΦM allows simultaneous targeted administration of an immune-related gene and chemotherapeutic drug. Subsequently the pH-sensitive-modified DOX is created employing the standard Schiff base reaction among DOX and partially-oxidized hyaluronic acid (OHA). In the meantime, to improve the rate of transfection of the shRNA-Ptpn2 plasmid, tumor-homing peptides iRGD and branching poly-ethylenimine (PEI) were employed to compact shRNA-Ptpn2 and create a complex (shRNA-PEI-iRGD, RPR) as shown in Figure 7B. This RPR surface is electrostatically coated with HA-DOX (HD), resulting in HD@RPR. Finally, polarized M1-membranes were employed to conceal HD@RPR. The shRNA-Ptpn2 system linked with DOX acts as an immuno-combined-chemotherapy and enhances the overall number of CD8+ T cells while inhibiting the development of primary melanoma rather than affecting the surrounding tissue 82.

### Anti-HER2 and immunization

The human epidermal growth factor receptor 2 (Her2), which is overexpressed in breast cancer, has emerged as a potential therapeutic target. Affibodies are small, robust proteins engineered to bind to a large number of target proteins or peptides with high affinity. They can attach to the HER2 receptor on cancer cells, just like the drug trastuzumab (Herceptin)**,** but they bind to different parts of HER2 83. Targeted therapy and its outcome for this subtype are inadequate, resulting in a low survival rate 84. Adjuvant chemotherapy, such as HER2+ antibody drug-conjugates (HER2+ ADCs), is now the primary treatment option for HER2+ breast cancer 85. The Xinan team developed a genomic reprogramming MΦM-encapsulated payload nano platform for HER2+ cancer treatment using co-assembly of PLGA-NPs and modified MΦM. Near-infrared (NIR) fluorescent dye ICG or DOX was introduced over PLGA cores, and anti-HER2 affibody persistently expressed on MΦM. Compared to NPs with typical MΦM coating, ICG/DOX@AM-NP armed with anti-HER2 affibody demonstrated outstanding HER2-targeting capabilities and exhibited synergistic suppression of HER2+ cancer cells by triggering apoptosis and inhibiting the PI3K/AKT signaling pathway 86. Zhiqiang et al. constructed PEI-MM-PLGA-DP/OVA to explore the macrophage immunomodulatory function of PEI-modified MΦM-coated PLGA-NPs containing Dendrobium-devonianum-polysaccharides. PEI-MM-PLGA-DP/OVA boosts antigen absorption by macrophage and lymphocyte proliferation, thereby increasing MHC II, CD80, and CD86 expression levels in immunized mice 87.

PsEUL belongs to biological macromolecules isolated from Eucommia ulmoides leaves. Biomimetic PsEUL-PLGA-NPs covered with MΦM (MΦM-PPsEUL) enhanced the phagocytic efficiency of macrophages without changing their activity, significantly enhanced the immunological organ index of mice, and strengthened the proportion of ovalbumin-specific IgG-antibodies in serum. The expression level of IFN-γ and IL-4 cytokines as well as the expression levels of NF-κB p65, TRAF6, MyD88, and TLR4 proteins elevated in the mice's spleen. MM-PPsEUL also displayed therapeutic influence on the inguinal lymph nodes and delayed the antigen release time, thereby producing a protracted immune response 88. Engineered and implanted vaccination generated from macrophages prevents postsurgical tumor recurrence. The vaccine contained hybrid membranes comprised of macrophages and tumor cells, as well as an immunoadjuvant called cytosine phosphate-guanosine-oligo-de-oxy-nucleotides (CpG ODNs). This vaccine was then incorporated into a calcium alginate hydrogel for tissue localized distribution, which enhanced systemic immunity by promoting DC maturation and memory T cell stimulation, resulting in a self-supplying circulation in TME. The multifunction vaccine made from biomacromolecules and naturally derived materials is a biocompatible and adaptable tool for avoiding postsurgical tumor recurrence 89. When combined with checkpoint inhibitors, macrophage-mediated and light-triggered precise delivery of cytotoxic drugs generates "*in situ* vaccines" resulting in increased chemo/photo/immunotherapy of primary and metastatic breast tumors 90.

### Inducing immunological cell death

Immunological cell death (ICD) is a specialized form of regulated cell death that not only leads to the elimination of cells but also actively stimulates an immune response against antigens released from the dying cells and serves as a bridge between the dying cells and the immune system. Chuan et al. introduced DOX into BSA-protected gold-nanoclusters (B-AuNCs), resulting in small B-A (D) structures. B-A(D), which penetrates deep into the tumor, forms a corona by adsorbing onto the surface of (C/I)BP, resulting in a (C/I)BP@B-A(D)-NPs. M1-macrophages were employed for coating (C/I)BP@B-A(D)-NPs, called (C/I)BP@B-A(D)&M1-macrophage and administered in combination with photodynamic therapy (PDT) to induce antitumor immunity, destroy localized malignant cells by generating ROS, and improve antitumor immunity through boosting tumor-derived antigen absorption and presented by DCs to T cells. (C/I)BP@B-A (D) &M1-macrophage along with the laser treatment group, DOX, and photosensitive Ce6 were administered to induce efficient direct tumoricidal effects as well as tumor cell ICD. Thus, the significant ICD induction by (C/I)BP@B-A(D)&M1-macrophage with Laser therapy promotes DC development which leads to strong immune response targeting endogenous tumor antigens *in vivo* 91. Integrated-based therapy M1/PLGA@IR780/CAT-NP, designed by the Cheng team combines synaptic therapy (SDT) immunotherapy along with immune checkpoint inhibitors targeting PD-L,1 developed by coating PLGA-NPs with M1-MΦM and loaded with catalase (CAT) and IR780. M1/PLGA@IR780/CAT in conjunction with US effectively triggers the maturation of tumor-draining lymph node DCs. The CD80+CD86+ DC ratio was quite 32.4% in the M1/PLGA@IR780 +US group, whereas the M1/PLGA@IR780/CAT + US group exhibited the highest proportion, 52.8%. The results were substantially greater than those for M1-nanovesicles alone (18.2%) and M1/PLGA@IR780/CAT alone (19.8% and 19.0%). Thus, after M1/PLGA@IR780/CAT disrupted tumor tissue via SDT, tumor-associated antigens were transmitted to nearby lymph nodes under the control of M1-nanovesicles, facilitating the transformation of immature DCs to mature, associated with the cellular results of bone marrow-derived dendritic cells (BMDC) maturation 66. TAM-coated NPR@TAMMs by synthesizing rare earth up-conversion nanoparticle (UC-NP)-based photosensitizers, called NaYF4: Yb, Er@NaYF4 conjugated with Rose-Bengal (NPR) with minor modifications. These NPs convert NIR light irradiation to visible light emission and serve as a potential "nanotransducer" for deep tumor therapy. The TAM-membrane (TAMM) was subsequently produced from purified primary TAMs separated by anti-F4/80 and CD206 beads and coated on NPR (NPR@TAMM). TAMM developed from a primary tumor with distinct antigen homing affinities and immunological compatibility. TAMM reduces the macrophage colony-stimulating factor 1 (CSF1) released by tumor cells in the TME, preventing the interaction between TAM and cancer cells and inducing ICD 45. Artificially modified macrophages exhibit remarkable potential as regulated drug reservoirs and ICD-inducing vehicles for achieving synchronized chemo/photo/immunotherapy of both primary and metastatic cancer. Yanjuan et al. developed Oxa(IV)@ZnPc@M mediated chemo-PDT coupled with anti-PD-L1, which can successfully destroy primary and bone metastatic tumors 90. MΦM-camouflaged nanoparticles M@PFC effectively deliver CpG into the tumor. PF3-PPh 3 enhances significant ROS generation and triggers ICD in tumor cells when exposed to light. The synergistic effects combining PDT features associated with the aggregation-induced emission (AIE)-active photosensitizer with immunotherapy attributes CpG considerably delays tumor recurrence following surgery 92. A polycationic carrier coated with MΦM biomimetically created from endogenous spermine monomers via diselenide linkages. The designed Trojan horse delivery vehicle (MPM-camouflaged nanoplexes) exhibits ideal compression effectiveness for siRNA oligo against PD-L1 (siPDL1) along with intracytoplasmic release features resulting from its sequential breakdown driven by redox milieu in tumor cells. In addition, photosensitizer co-loading generates ROS in response to light irradiation, which speeds up carrier degradation and cargo release while improving PD-L1 blockage-mediated immunotherapy by inducing *in-situ* ICD 93.

## Gas therapy

Gas therapy is a therapeutic modality that utilizes gases or gas mixtures such as carbon monoxide (CO), nitric oxide (NO), and hydrogen sulfide (H_2_S) to target cancer cells 94. Over the past few years, cell membrane-based biomimetic NPs attracted great attention in therapeutic applications. Chunai and his colleagues developed a hybrid-biomimetic membrane camouflaged core-shell nanoplatform to deliver metformin (Met) and siFGL1, respectively. Met and siFGL1 were enclosed in PLGA to form the core, covered with a hybrid-biomimetic membrane made up of macrophages and cancer cells to generate a multi-targeting biomimetic nanoplatform. MC-PLGA@Met-CO_2_/siFGL1-NPs represent a pH-triggered CO_2_ gas generating nanoplatform designed to improve endosomal/lysosomal release of encapsulated siRNA for effective cytosolic siRNA delivery. Met's guanidine group reacts reversibly with CO_2_ to produce Met-CO_2_, allowing for pH-dependent CO_2_ capture/release, facilitating encapsulated siRNA escape via low pH-activated endosomal/lysosomal mechanisms. This hybrid-mimicking membrane, created by combining RAW264.7-MΦM and 4T1-breast cancer cell membranes, demonstrated multi-targeting capabilities 95. After administration into a tumor-bearing mouse model, FBN@M specifically targets the tumor region following innate inflammation-directed chemotaxis of MΦM and NaHCO_3_ in response to acidic TME since the permeability of H^+^ inside generates CO_2_ to break up MΦM, leading to the release of BMS-202 and blocking the PD-1/PD-L1 pathway 52. At the same time, NONOate breaks down under precise irradiation circumstances, generating NO. The NO combines with superoxide anions to generate peroxynitrite anions (ONOO-), which are recognized for their powerful cytotoxic effects. This increases the therapeutic impact. Furthermore, the administration of perfluorohexane (PFH) improves the availability of O_2_ inside the TME. This innovative combination therapy, comprised of MΦM-coated liposomes containing IR780, NONOate, and perfluorocarbon (IR780-NO-PFH-Lip@M), substantially improved toxicity against breast cancer by generating both heat and reactive nitrogen species (RNS), resulting in a significant reduction in cancer cell growth and proliferation 96. The NIR light-controlled self-destruction of macrophage-based drug delivery systems (MMDM) resulted in a sufficient MDM release from the carrier cells. The released MDM causes O_2_ generation in TME via H_2_O_2_ breakdown. Mn^2+^ facilitates a Fenton-like reaction and converts intracellular H_2_O_2_ into highly reactive hydroxyl radical (-OH). As a consequence, loaded NP and NIR light-controlled self-destructive macrophages, with oxygen generation and GSH depletion capabilities, potentially act as a viable platform for synergistic treatment 97. Gold-NPs (Au-NPs) produced *in situ* on BMSNs (black-phosphorus quantum-dots (BPQDs) doped mesoporous-silica-frameworks), also known as Au-BMSNs, act as a type of SDT agent. The CO-releasing molecules CORM-401 were then inserted into Au-BMSNs (CAu-BMSNs), which have natural porosity features and function as a stimuli-responsive agent. Following wrapping MΦM over CAu-BMSNs, the biomimetic nano-system (N@CAu-BMSNs) equips tumors with active targeting capability and efficiently reduces tumor development by causing mitochondrial rupture and cell death through ultrasound-triggered CO and ^1^O_2_ production. In addition, the SDT/CO treatments lead to considerable immunogenic death of tumor cells and long-term immunological memory 98. NIR-responsive MΦM-camouflaged cascades CuS-based nano-systems delivered GOx and catalyzed (CAT) to tumor areas. GOx was created to deprive tumors by consuming glucose. Furthermore, H_2_O_2_ catalytically generated by GOx and endogenous H_2_O_2_ decomposing *in situ* by CAT produce sufficient oxygen to increase the effectiveness of the starvation therapy and effectively alleviate the lack of O_2_ in TME, as well as lower the pH value of TME, which promotes chemodynamic therapy (CDT) 99. TK-M@Man-HMPB/HCQ when encountering a high concentration of H_2_O_2_ in TME, thylakoid (TK) membrane catalyzed H_2_O_2_ to generate O_2_ for hypoxia alleviation. Overall, the constructed hybrid-membrane comprised of macrophage and TK contributes to effective tumor-targeting distribution, localized rupture of the membrane along with regulated NPs release, and hypoxia alleviation of TME 70. Liposomes wrapped with MΦM, comprising macrophage-associated membrane proteins, demonstrated potential in biomimetic delivery systems for targeted tumors while retaining their innate tumor-homing features. The IR780-NO-PFH-Lip@M constructed biomimetic delivery system incorporated IR780, NONOate, and perfluorocarbon. This designed encapsulation is intended to create a synergistic combination of PDT and reactive-nitrogen-species (RNS) therapy. Under NIR laser irradiation, IR780 generates ROS such as superoxide anion (O_2_^•-^), singlet oxygen, and OH. Under laser irradiation, NONOate produces NO gas, interacts with IR780-induced ROS and forms peroxynitrite anion (ONOO-), leading to programmed cell death in tumor cells 96.

Reactive CaO_2_-NPs (core) are isolated by biocompatible zeolitic imidazolate framework-8 (ZIF-8) doped with Fe^2+^ (shell), and then encapsulated by MΦM to make CaO_2_@Fe-ZIF-8@MΦM (symbolized as CFZM) exhibit active tumor-homing by MΦM coating, TME-responsive cargo release, self-supplied H_2_O_2_ for fostering Fenton-like reactions, and effective generation of toxic-OH in CFZM and high-efficacy tumor destruction in BALB/c mice bearing CT26 tumor cells 100. Surface-engineered chlorella (Chl, a type of green algae) acts as a targeted-drugs carrier and long-term O_2_ provider (by photosynthesis) for significantly improved SDT through hypoxia relief and chloroquine-phosphate autophagy suppression. MΦM were coated onto Chl to create macrophage-mimetic Chl (MChl) for enhanced biocompatibility and targeted-tumor accumulation through MΦM inflammatory homing features. Additionally, membrane coating onto Chl enabled lipid insertion, resulting in β-cyclodextrin (β-CD) modified MChl (CD-MChl). In the meantime, supramolecular conjugates of MChl-NP were created through host-guest interactions among CD-MChl and adamantane (ADA) modified-liposome (ADA-NP), and anchored liposome accompanied CD-MChl within tumor tissue to co-deliver Chl, hematoporphyrin, and chloroquine phosphate (which is loaded in ADA-NP). MChl-CQ-HP-NP enables local oxygenation in melanoma 101.

**Figure 8:**
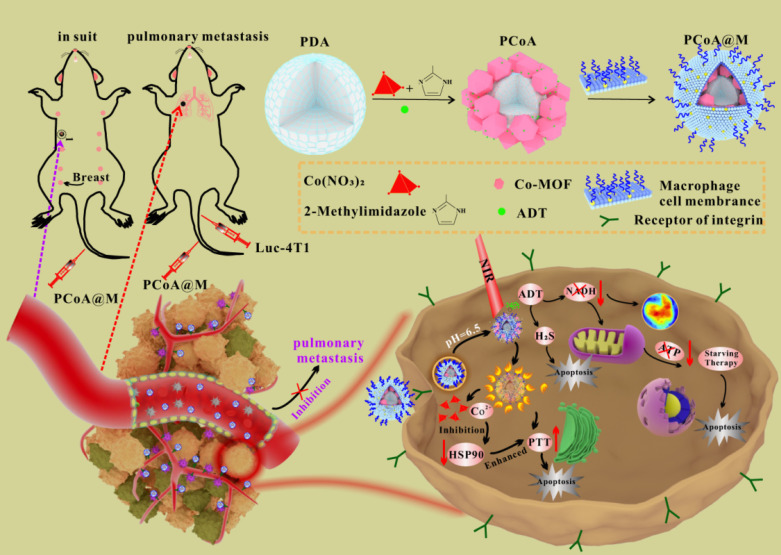
PCoA@M preparations PDA, ADT, and Co-MOF were prepared by a one-pot method and modified with MΦM to form PCoA@M and enriched in the TME via targeting integrin. PCoA@M enhanced PTT by blocking HSPs, degrading and releasing drugs under the TME acidic conditions, reducing NADH generation, and achieving PTT gas synergistic starvation therapy. Reproduced with permission 103. Copyright 2022, Nature.

NO-functionalized black phosphorus nano-sheets (BPA) develop via an esterification reaction between the carboxyl-group of L-arginine (Arg) and the hydroxyl-group (P-OH) developed from the preliminary oxidation on the surface of black phosphorus. Subsequently, glucose-oxidase (GOx) is introduced to Arg via amidation, resulting in a multi-modal nanodrug (BPAG) which induces the release of H_2_O_2_ along with NO through cascaded oxidation of glucose and Arg. MΦM is employed to coat NPs under ultrasonic conditions, allowing BPAG to effectively target tumors. The membrane-coated BPAG (M@BPAG) increases penetration of blood-brain barrier for glioblastoma targeting 102. PCoA@M biomimetic functional nanoplatform **(Figure 8)** with an MΦM is designed. Polydopamine (PDA) serves the core due to the strong chelation generated by its catechol groups to metal ions. The co-metal organic framework (co-MOF) forms the shell, resulting in a heterogeneous structure. Furthermore, anethole trithione (ADT), a H_2_S-releasing precursor, is inserted into the pores of MOF and gaps of heterostructure employing a one-pot approach. Ultimately, coating with MΦM enhances the nanocarrier's specificity as well as stability. The biomimetic nanocarrier is designed with specific functionalities. Upon Co-MOF degradation, significant release of loaded ADT occurs. ADT, serving as an H₂S donor, undergoes enzymatic conversion in breast cancer cells, generating high H₂S levels to suppress tumor growth and metastasis 103.

## Theranostics-based therapy

Theranostics integrates therapy and diagnostics into a single platform in cancer treatment. The term is derived from "therapy" and "diagnostics," emphasizing the dual function of diagnosing and treating diseases simultaneously or in a coordinated manner. The theranostic approach employs liposomes as a carrier, quaternary quantum dots (QDs) embedding hydrophobic ZAISe/ZnS-QDs in phospholipid bilayers, loading hydrophilic DOX in internal vesicles, and fusing with isolated MΦM. The quaternary ZAISe/ZnS QDs-based fluorescent imaging was employed not only to trace the spatial distribution of NPs but also to precisely localize tumors and guide chemotherapy *in vivo*. MΦM with α4-integrins enabled liposome-based super particles to attach VCAM-1 to cancer cells and escape immune responses. The incorporated liposome is predicted not only to recognize the tumor for imaging-guided cancer surgery but also to be employed as a chemotherapeutic treatment for pre- and post-surgery 104. Shichao et al. developed RIFe@TRM, composed of ICG, imiquimod (R837), and murine-derived 4T1 breast cancer cells with RAW264.7, effectively employing MRI for monitoring drugs' biological distribution throughout the therapy process. The RAW264.7 membrane considerably improves immune escape abilities, while the 4T1 cell membrane boosts targeting effectiveness for breast cancer and exhibits tumor-specific self-recognition, prolonging circulation time, and increasing *in vivo* targeting ability 61. HMFe@BS was developed by encapsulating virus-like hollow-mesoporous ferric oxide NPs (HMFe) with encapsulating 4-(2-aminoethyl) benzene-sulfonamide (BS), a CAI, and activated by physical and electrostatic adsorption. HMFe@BS is camouflaged with MΦM (MΦM@HMFe@BS) to evade macrophage-phagocytosis, increase blood circulation time, and accumulate within the breast cancer through the interaction between α4-integrin in MΦM and breast cancer overexpressed vascular cell-adhesion molecule (VCAM)-1. MΦM@HMFe@BS more specifically deteriorated within TME, Fe can be used in highly efficient MRI to monitor biodistribution and therapeutic progress 105. Tumor monitoring is achieved by employing hollow bismuth selenide (BS-NPs) with high absorbance of both X-ray and NIR light, which are effective for CT imaging (high resolution and easy three-dimensional visualization of tissues of interest) and infrared imaging (IRT). MΦM-camouflaged quercetin (QE)-loaded-hollow-bismuth-selenide-NPs (also known as M@BS-QE NPs) demonstrated exceptional therapeutic efficacy due to their ability to recruit CCL2/CCR2 and identify α4/VCAM-1 and inhibited heat shock protein 70 (HSP70) and down-regulated p-Akt/MMP-9 44.

Gold nanodendrite-based (AuND) nano-theranostic agents with multipurpose features were made through optimizing geometric configurations of AuND for achieving NIR-II localized surface plasmon resonance (LSPR), then modifying with mitochondria targeting compound (i.e., triphenylphosphonium (TPP)), inserting NIR-photosensitizer (i.e., ICG), and finally coating with MΦM to achieve synergistic NIR-I PDT and NIR-II photothermal therapy. The developed hybrid nano-systems of AuND-TPP-ICG@MΦM demonstrate a number of distinctive characteristics suitable for cancer theranostics, including potent absorbance and excellent photothermal conversion capability of AuND at NIR-II for PTT, achieving extended tissue penetration and maximum permissible exposure, MΦM-coating for specific accumulation in cancerous cells and photo-responsive release of ICG, TPP alteration for mitochondria-targeting while providing SERS-based Raman imaging reporter and multimodal image-guided therapy (NIR-II PAI, fluorescence (FL) imaging 106.

**Figure 9:**
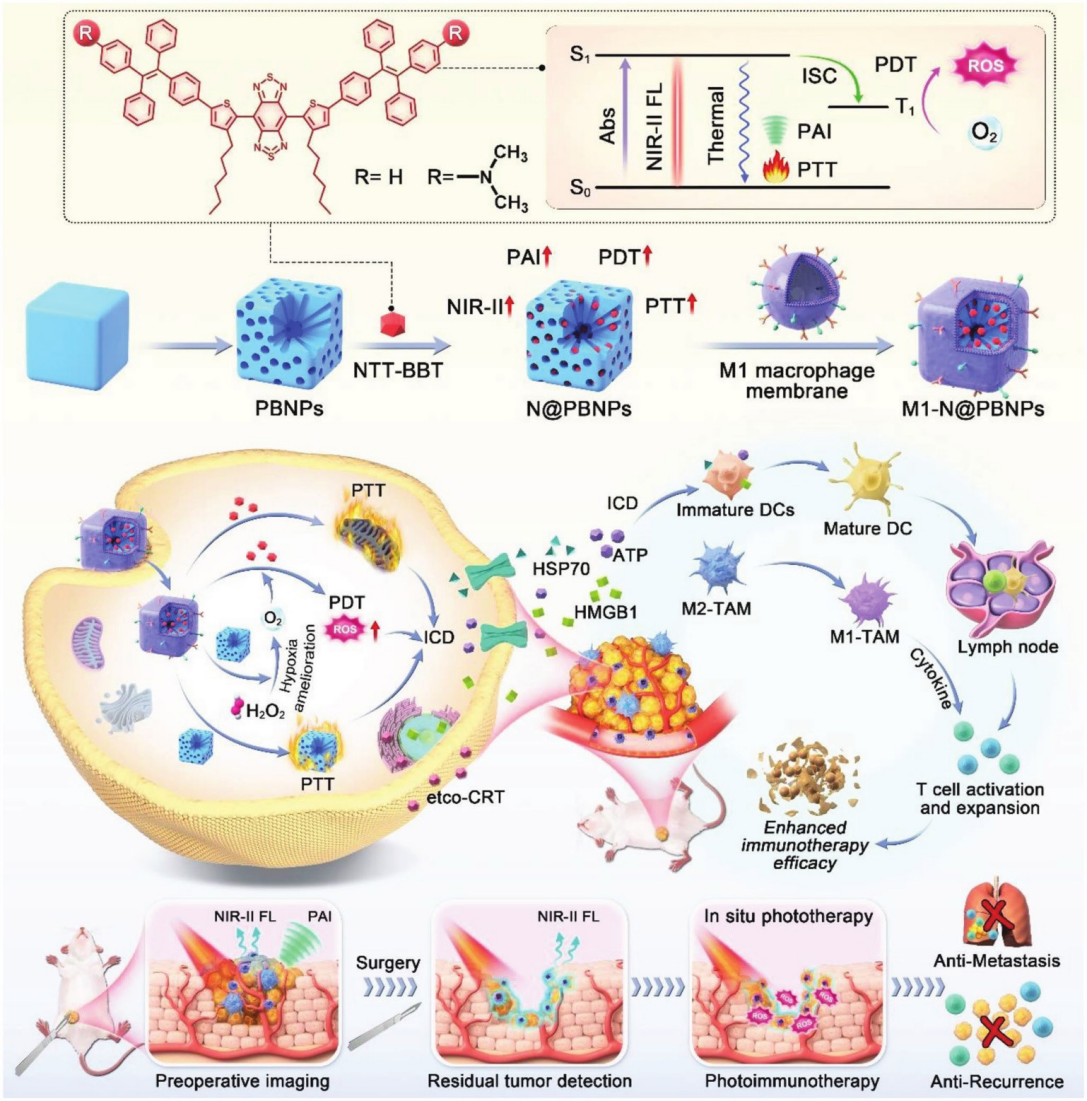
Schematic illustration showing the integration of NIR-AIEgen with mesoporous PB nano-catalyzer boosts the theranostic performance for NIR-II fluorescence and PA imaging-guided robust cancer immunotherapy. Reproduced with permission 107, copyright 2024, Wiley online library.

Water-dispersed Fe_3_O_4_-magnetic nanoclusters (MNCs) with adjustable sizes were produced using polyethyleneimine (PEI) to serve as a surfactant. The positive electrical charges on the clusters were then exploited to attach siRNA via electrostatic interactions. The MNC:siRNA combination was further camouflaged with MΦM. Endogenous macrophages identified the magnetosome as a self-partner, prolonging its circulation in the blood. MΦM can be pre-engineered with azide by intrinsic biosynthesis and metabolic adoption of phospholipids. This approach cut down the path for future decorating with tumor-targeting peptide Arg-Gly-Asp (RGD) employing easy-to-follow click chemistry. The developed platform demonstrated improved advantages in programmed siRNA administration, such as prolonged circulation duration, MR imaging, enhanced tumor accumulation, higher tumor uptake, and positive intracellular fate 53. **Figure 9** depicts an innovative approach for advanced cancer immunotherapy incorporating AIEgens into a Prussian-blue (PB) nanocarrier to enhance theranostic properties and subsequently encapsulated in M1-MΦM to increase accumulation at the tumor spots. Furthermore, PB demonstrated catalytic ability to react with tumor-overexpressed H_2_O_2_, producing O_2_ *in situ* and facilitating light-triggered ROS generation. The PB's NIR absorption characteristics further enhanced the PTT effect. As a result, the combination of PB nanocarrier with AIEgen produced a high-performance phototheranostic platform with precisely tailored characteristics. When exposed to NIR light, it demonstrated not only NIR-II fluorescence and photoacoustic (PA) imaging features but also a robust ability to induce ICD in tumor cells and provided valuable diagnostic information through NIR-II fluorescence and PA imaging, facilitating precise tumor delineation and providing guidance for subsequent photoimmunotherapy 107.

**Figure 10:**
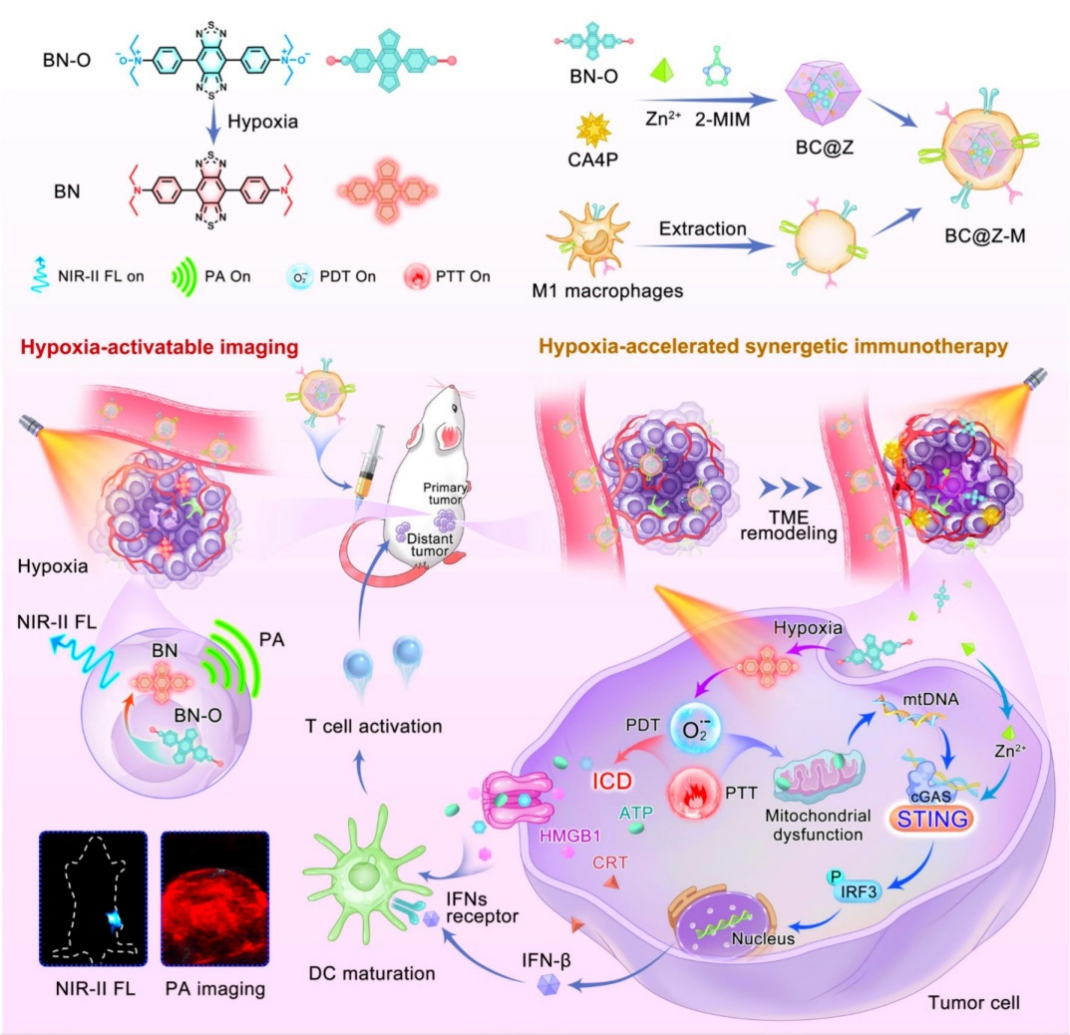
The illustration demonstrates that the hypoxia-activated nanoplatform integrates NIR-II fluorescence (NIR-II FL) and photoacoustic (PA) imaging for precise tumor localization, along with synergistic immunotherapy via TME remodeling, ICD, and STING pathway activation to enhance cancer treatment. Reproduced with permission 110, copyright 2024, Nature.

Development of a biodegradable nanoplatform with deep tumor penetration for combination therapy against metastatic breast cancer. Nano-engineered hollow and mesoporous-polycrystalline CuSNPs were surface-loaded with paclitaxel (PTX) and nano-cloaked with MΦM to generate PTX@CuS@MΦM-NPs. Following exposure to NIR, the polycrystalline nano-constructs disintegrated into ultra-nanocrystals, resulting in excellent biodegradability and nontoxicity. Co-administration of iRGD increased tumor vascular permeability. Furthermore, iRGD's affinity for 4T1 improves the penetration of PTX@CuS@MΦM-NPs, allowing them to penetrate the tumor vasculature and subsequently undergo internalization via α4-VCAM-1 interactions. After the tumor engulfs PTX@CuS@MΦM-NPs, the membrane cloaking acts as a barrier, preventing CuS and PTX from being released for their respective photo and chemotherapy behaviors. As a result, the optimal membrane-escape strategy provides additional specifications for the biomimetic nano-system following internalization. After NIR laser irradiation, the proton sponge effect causes MΦM to erupt, releasing CuS (PTT/PDT) and PTX (chemo) for a combined therapeutic effect 108. CDM@MUiO-DP@MCHM is a mitochondria-targeted therapeutics platform constructed from encapsulated MUiO-66 metal organic-frameworks (MOFs) coated by macrophage cancer hybrid membrane (MCHM) and incorporates a microRNA (miRNA) biomarker detection probe (DP) for diagnosing cancer and suppresses cancer growth by depleting mitochondrial copper. The encapsulation of MCHM following intravenous injection not only enhances the cancer-homing targeting ability of NPs, but also endows NPs with immune escape potential in order to prolong circulation time. The fluorescent signal identified the miRNA-21 biomarker for diagnosis, whereas the copper-depleting moiety (CDM) caused energy scarcity and damaged the mitochondria membrane potential, resulting in apoptosis of cancer cells 109. Synthesis of a molecular probe, designated BN-O, derived from an N-oxide framework, capable of transitioning from an "A-A" to a "D-A" configuration, as shown in (**Figure 10),** facilitating an activated NIR response with substantial NIR-II fluorescence/photoacoustic signals. In addition to the activation of imaging signals, BN-O demonstrates hypoxia-induced type-I PDT and PTT effects. A hydrophilic molecular probe, together with a vascular disrupting drug, is encapsulated inside an acid-degradable MOF nanocarrier and further cloaked with M1-like MΦM to create a tailored theranostic nanoplatform. The vascular disrupting drug alters the TME, therefore limiting the tumor's nutrient and oxygen supply while enhancing hypoxic TME to enhance hypoxia-activated phototherapy. The nanoprobe effectively illuminates hypoxic tumors *in situ* by generating strong turn-on NIR-II fluorescence and photoacoustic signals, enabling precision and providing essential guidance for future photoimmunotherapy. This study presents novel high-efficient and activatable theranostic procedures designed for accurate image-guided tumor immunotherapy 110.

## Biomolecule-conjugated macrophage membrane

Tailored nanomedicine specifically targets cancer cells through their interaction with overexpressed receptors on their surfaces, hence improving target availability and preciseness. Saikosaponin-D (SsD), a triterpene saponin produced by Bupleurum, has potential therapeutic qualities for cancer treatment. However, SsD's non-specific distribution and poor pharmacokinetics led to significant side effects and systemic toxicities, which were restricted in clinical studies. To overcome this limitation, Kaiju et al. developed SCMNPs delivering drugs that mimic macrophages by coating MΦM with T7-peptide on the outermost layer of PLGA nanoparticles. Surprisingly, SCMNPs displayed targeted selectivity to cancer cells with features of immune escape, preferential accumulation, and increased cell endocytosis, and successfully reduced tumor growth and metastasis. SCMNPs achieve maximum therapeutic efficacy with minor adverse effects via triggering the angiogenic pathway, indicating great promise for a precise and successful therapeutic approach 111. Haiqiang et al. developed MΦM coated emtansine liposome (MEL) by isolating MΦM from RAW 264.7 cells with an elevated level of α4 and β1-integrins, then enclosed cytotoxic anti-cancer drug emtansine in pH-sensitive liposome and coated it with the isolated MΦM to create MΦM-coated emtansine liposome MEL for targeting metastatic sites. Emtansine liposomes decorated with MΦM exhibit selective metastasis targeting and anti-metastatic efficacy in a breast cancer model 112.

Biomimetic system for delivering drugs based on MSN for immune evasion and targeting of tumors. Co-extrusion of folic acid-modified mesoporous silica FMSN cores with lipid hybridized MΦ resulted in FMSN@MΦM retaining important proteins such as Integrin α4 and Integrin β1. This reduces the clearance of FMSN@MΦM by phagocytes *in vitro* and efficiently inhibits tumor cell growth. The immune-escape and tumor-targeting capabilities of FMSN@MΦM an innovative bionic drug carrier, possibly give more alternatives for anticancer treatment 113. TAMs membrane-camouflaged pH-responsive DOX loaded with hyaluronic acid (HA)-g-poly (histidine) polymeric micelles (DHP@M2's) exhibited high levels of α4β1 integrin, allowing for dual-targeting of CD44/VCAM-1, promoting intracellular DOX accumulation and effective ICD induction 78. RAW 264.7 cells exhibit substantial levels of integrins α4 and β1, and offer great affinity for VCAM-1 of 4T1. Nano-engineered hollow and mesoporous polycrystalline CuS-NPs preloaded with PTX were nano-cloaked with MΦM to form PTX@CuS@MM-NPs, combining the administration of iRGD and increased tumor vascular permeability. Furthermore, the affinities of iRGD for 4T1 increase the permeability of PTX@CuS@MM-NPs into the deep tumor site. With the prolonged blood circulation time and chemotactic tendency (of MΦM) for persistent inflammatory responses 108.

JQ1, an inhibitor of bromo-domain-containing protein 4 (BRD4), and celecoxib, an inhibitor of cyclooxygenase-2 (COX-2), were co-loaded into chondroitin sulfate (CS) to form CS@JQ1/CXBNPs. The biomimetic nanoplatform MM@P3 further covers the branching polymer poly(β-amino-ester) self-assembling NPs with melittin-anchored MΦM. The CS@JQ1/CXB and MΦM@P3NPs demonstrated high immune activation efficiency. The combination therapy demonstrated synergistic toxicity and anti-migration ability in breast cancer tumor-bearing mice by stimulating tumor immune response and reducing angiogenesis 114.

**Figure 11:**
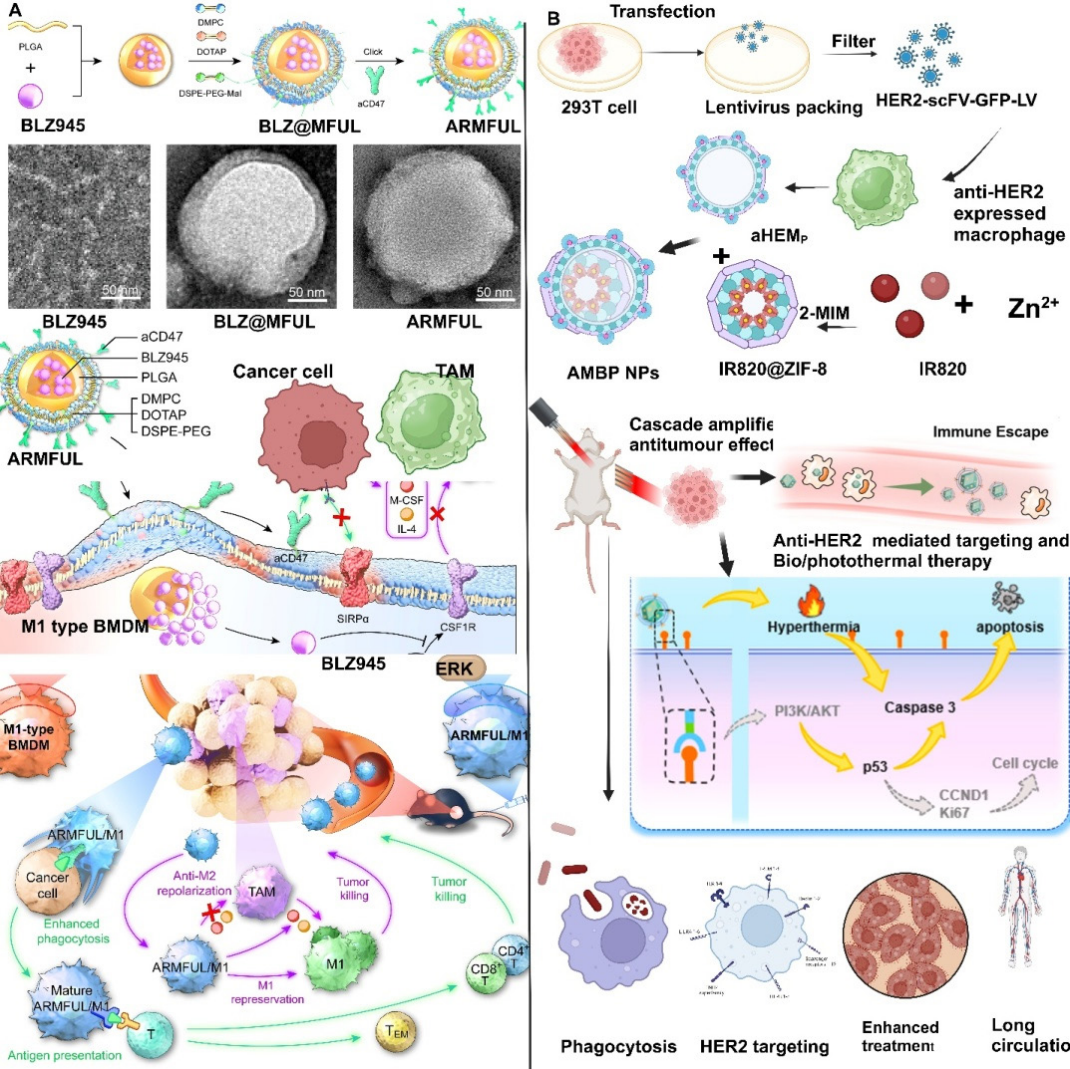
**Macrophage membrane conjugated biomolecules and immunization (A)** Schematic illustration of the preparation of ARMFU. M1-macrophages engineered with ARMFUL and CSF1R-inhibitor BLZ945 were loaded in the PLGA-based polymeric core, and aCD47 was conjugated on the fusogenic lipid shell surface, showing in the TEM image a aCD47, BLZ@MFUL, and ARMFUL. This ARMFUL can fuse with the M1-MΦM to simultaneously insert aCD47-modified lipid shells on the surfaces directly and release the BLZ945-loaded core into the cytoplasm, formulating ARMFUL/M1 for back-transfer and could remodel the tumor microenvironment. ARMFUL/M1 macrophages could remodel the TME, activate T-cell cytotoxicity, and induce systemic immunological memory to synergistically inhibit tumor growth. Reproduced with permission 80. Copyright 2023, science. (B) Illustration of antiHER2-engineered macrophage biomimetic photothermal (AMBP) systems for photothermal/biotherapy of cancer. Reproduced with permission 116. Copyright 2024, Elsevier.

Engineered macrophages were loaded with legumain-specific pro-peptide melittin (legM) and redox-sensitive prodrug of cytotoxic soravtansine (DM4) on the MΦM, resulting in legM and DM4-laden MDS. Legumain is an asparaginyl endopeptidase and is extremely active in TME. Living LD-MDS are actively attracted to tumor areas and subsequently change into DM4-loaded exosome-like nano-vesicles (DENs), which are taken by metastatic 4T1 cancer cells and induce significant immune response 115. M1 macrophages engineered with anti-phagocytosis blocking repolarizing resistant membrane fusogenic-liposome (ARMFUL) exhibit a core-shell structure designed for enhanced therapeutic efficacy **(Figure 11A)**. The core consists of a CSF1R-inhibitor BLZ945 inserted in a PLGA-based polymeric core and an aCD47 conjugated on the fusogenic lipid shell surface. This ARMFUL can merge with M1-MΦM, inserting an aCD47-modified lipid-shells directly onto the surfaces and releasing a BLZ945-loaded core into the cytoplasm, resulting in ARMFUL/M1 for back transfer. Surface presenting aCD47 protein enhances macrophage phagocytic abilities toward malignancies by inhibiting anti-phagocytosis CD47 proteins on tumors. Whereas, BLZ945 in the cytoplasm efficiently blocks intracellular-tyrosine-kinase of CSF1R and the resulting M2 polarization signaling pathway, allowing M1 macrophages to repress polarization to tumor-promoting M2-phenotype for long-lasting therapeutic effect 80. The engineered macrophage cell membrane of AMBP NPs escapes from the macrophage of the reticuloendothelial system and aggressively targets expressing 4T1 tumor cells, leading to enhanced accumulation in tumors with lower toxicity and enhancing the anti-tumor features of biotherapy, **(Figure 11B)** 116.

## Limitations and outlook

This review emphasizes the recent advancement of MΦM-based nanotherapeutics for targeted cancer therapy. Given the MΦM coating, nanotherapeutics are non-immunogenic, evade the immune system, and increase drug payload circulation time in the blood, maintaining natural tumor-homing ability and excellent biocompatibility. This allows for effectively targeting and penetrating both primary and metastatic tumor sites.

MΦM-camouflaged NPs combined with features of both natural or synthetic NPs and unique cell biological activities in one nanoplatform can substantially enhance their performances and present the abilities for single or multiple therapeutic agent encapsulation. While stabilizing and supporting morphological nanostructures, biomimetic delivery systems coated by MΦM possess camouflaging features *in vivo*, which significantly reduce opsonization, avoids clearance and selectively target tumor spots with suitable drug dosage. Encapsulating NPs with MΦM shows negligible effect on the cell membrane's biological activity or the NPs' properties. Furthermore, coated MΦM may erode under varying physiological conditions, accelerating the release of loaded drugs from NPs and providing a drug delivery system for synergistic or combination therapies. MΦM-based nanotherapeutics improve cancer therapy by improving selectivity, targeting, and safety. They can also be combined with many other drugs for synergistic benefits, making them potential strategies in targeted cancer therapy.

Despite their unique advantages, the main challenges concerning MΦM in the successful clinical translation of MΦM-coated NPs are its novelty and early stages, including high preparation complexity, heterogeneity of cell sources (e.g., gender, age, and health conditions), potential epigenetic modification during isolation and purification procedures, immunogenicity, poor consistency, large-scale manufacturing, and safety concerns regarding the possibility of coating techniques damaging the integrity and structure of membrane proteins and compromising the bio-functionality of MΦM 46,117. Moreover, obstacles that must be addressed include a lack of knowledge of the triggering mechanisms of macrophage migration and polarization, as well as the high complexity of the immune response inside the TME 118. Developing MΦM on a therapeutic scale for routine use is challenging due to immunological and safety issues concerning the presence of proteins that activate immune responses on cell membranes (e.g., MHC molecules). As a result, due to the higher possibility of immunological rejection when using allogeneic cells, human macrophages need to be genetically modified after extraction to avoid unwanted side effects 119. The absence of standard procedures for MΦM extraction and purification might lead to inconsistent results between batches. Ensuring repeatability throughout batches is highly difficult, not only because WBCs can undergo variations in their gene expression during *in vitro* treatment, but also because their functions fluctuate depending on the source 117.

MΦM-coated NPs exhibit a promising design for tumor targeting and controlled drug release, obstacles such as tumor heterogeneity, penetration efficiency, and release regulation under fluctuating conditions (e.g., pH variations in the endosome or extracellular matrix) limit their overall therapeutic effectiveness. Furthermore, long-term stability and integrity of MΦM-coating in the systemic circulation, together with possible immunological responses, provide considerable challenges for clinical use 42. M0-macrophages were selected because of their significant infiltration rate in breast cancer, however, macrophages are recognized for their phenotypic flexibility. Within the TME, macrophages polarize into M1 (pro-inflammatory) or M2 (pro-tumor) phenotypes, potentially influencing the stability and reliability of the platform's performance. The stability of MΦM in circulation or under oxidative stress, such as that caused by glucose oxidase activity, restricts their functional durability and effectiveness 59. Clinical studies may be limited by low monocyte or macrophage production. Unlike immortal and continually proliferating cell lines, monocytes have a 20-h blood half-life and evacuate the cell cycle after 7-10 days of proliferation and differentiation. Macrophages generated from monocytes or tissue lavage are short-lived, difficult *in vitro* proliferation, and difficult to genetically manipulate 120,121.

Furthermore, bone-marrow-derived macrophages (BMDMs) enhanced phagocytic activity and the highest proliferative capability relative to macrophages derived from the spleen or peritoneal cavity. Still, employing BMDMs in experimental research is challenging owing to their phenotypic and functional instability *in vivo* 122. Macrophage-derived exosomes coated with murine RAW264.7 exhibited enhanced blood circulation duration and targeted organ affinities, delivering effective chemotherapy to breast cancer cells 123. However, macrophage uptake makes extracellular vesicles susceptible to rapid removal from the bloodstream after systemic delivery. The ultimate half-life of EVs is a maximum of 60 minutes, and less than 5% of the administered dosage of exosomes persisted in the bloodstream at 3 hours post-injection 124.

Due to the unique features of MΦM-coated NPs, further developments in cancer-targeted therapy should be envisioned as MΦM biomimetic systems becoming more efficient and selective to tumors. Given these nano-systems immense potential to change therapy and diagnostics of numerous diseases in the future, the above limitations must be addressed immediately before employing them in clinical practice.

## Figures and Tables

**Figure 2:**
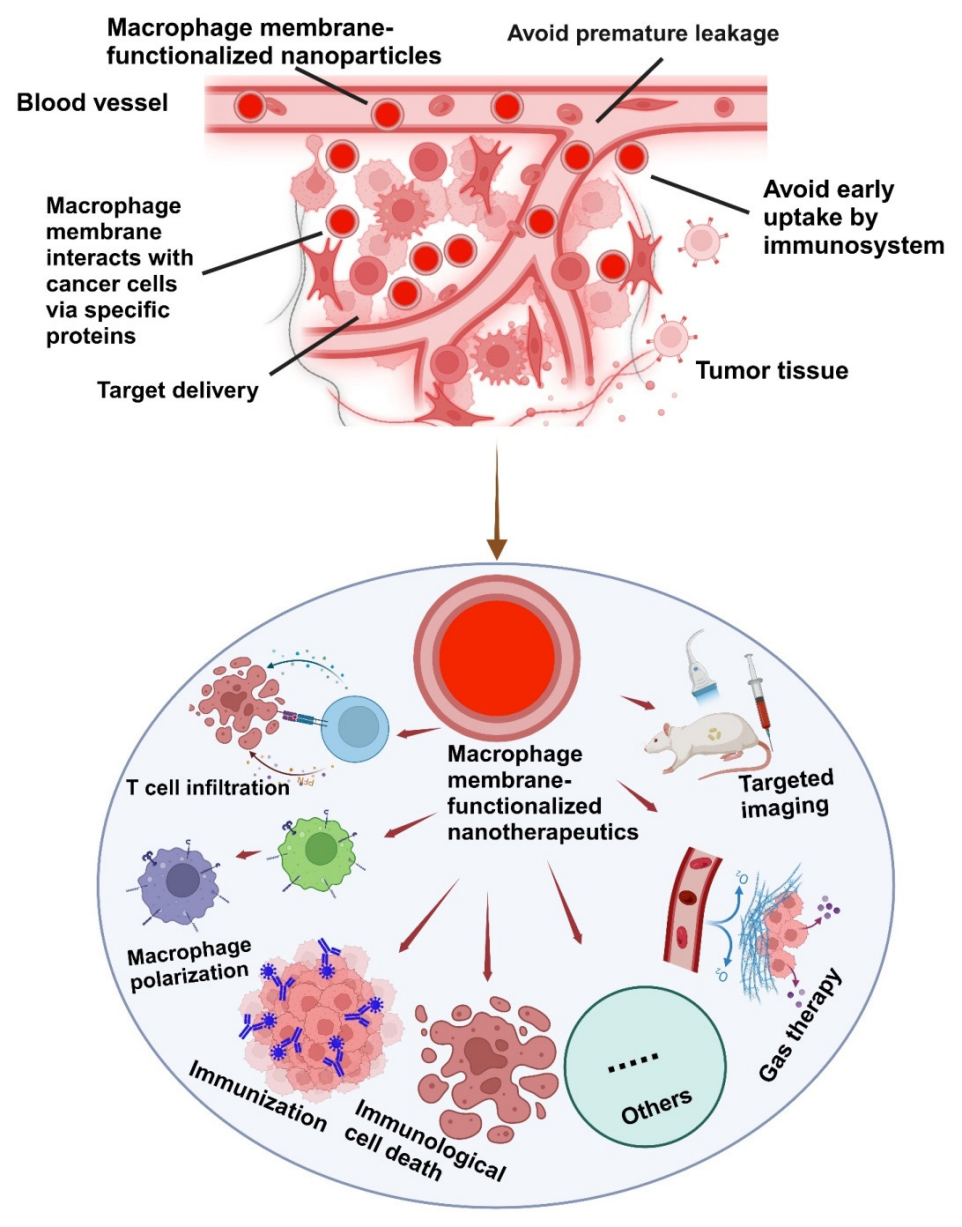
Schematic illustration of the possible mechanisms of MΦM-coated nanotherapeutics exhibiting high tumor-targeted delivery efficiency. Their inherent membrane characteristics enable effective tumor targeting, evasion of the immune system, avoidance of premature leakage, and enhanced cellular absorption improving therapeutic effectiveness while reducing off-target effects and systemic toxicity in tumor-targeted therapy.

**Figure 5:**
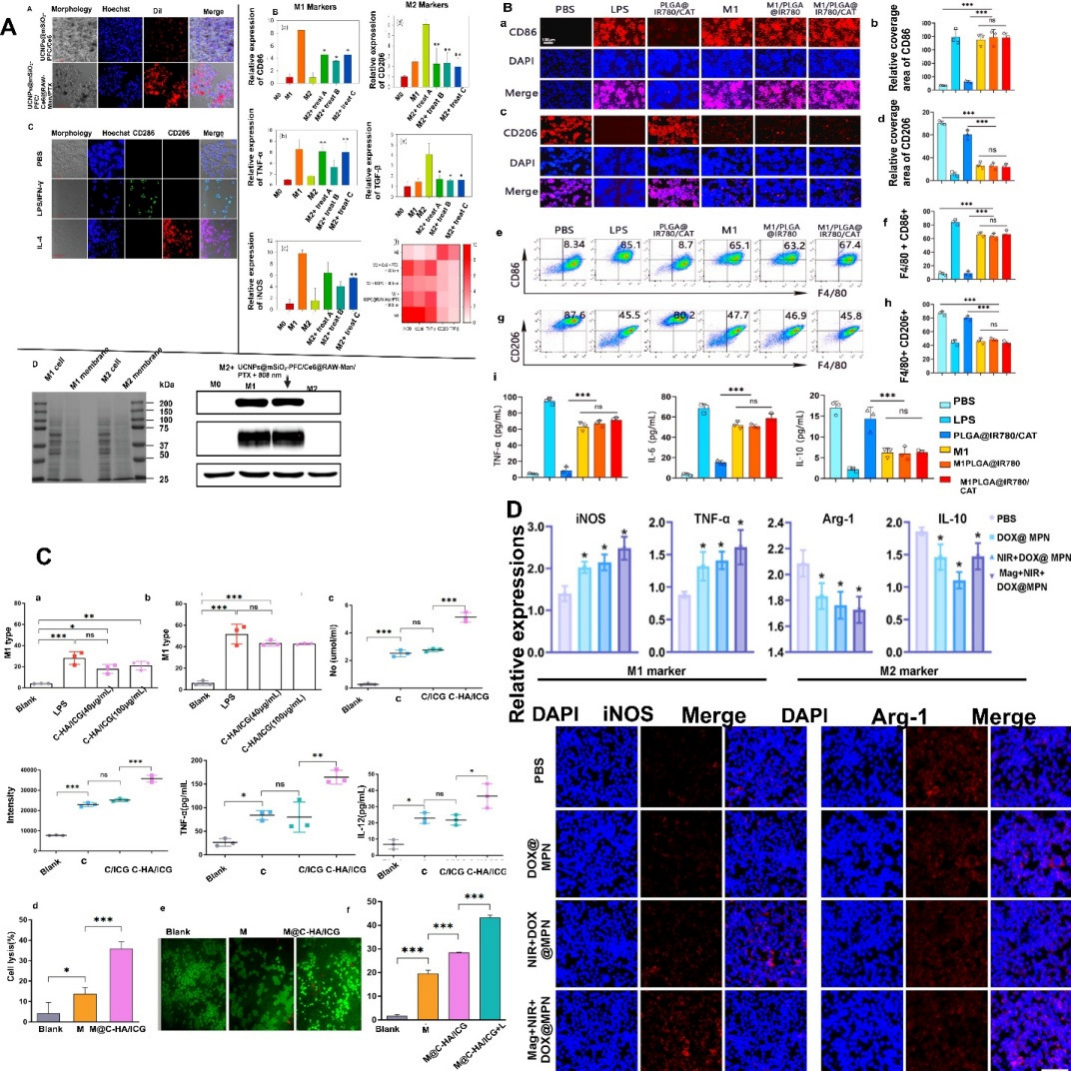
Targeting tumors and polarizing M2 tumor-associated macrophages to M1 phenotype (A) CLSM images of M2 RAW 264.7 cells after treatment with DiI-stained UC-NPs. (B) Relative expression of M1-markers [(a) CD86, (b) TNF-α, and (c) iNOS] and M2 markers (d) CD206 and (e) TGF-β as shown by qRT-PCR: Treatment A = Ce6 + PTX + 808 nm; B = UCNPs@mSiO_2_-PFC/Ce6 + 808 nm; C = UCNPs@mSiO_2_-PFC/Ce6@RAW-Man/PTX + 808 nm. (f) Relative expression levels of M1 and M2 markers. (C) CLSM images of RAW 264.7 cells treated with PBS, LPS/IFN-γ, and IL-4 in normoxic conditions showing CD86/CD206 expression. (D) Flow cytometry analysis of RAW 264.7 cells for M1, M2, and M2 + Treat =C. (E) Representative image of SDS-gel of M1 and M2 types (a) and western blot for cell membrane markers for M0, M1, M2, and M2 + Treat=C (b). Reproduced with permission 58. Copyright 2023, Elsevier. (B) Immunological regulations of M1/PLGA@IR780/CAT. Representative immunofluorescent staining images: red of M1-marker, CD86 (A) and M2-marker CD206 (C), and blue (DAPI). (B) The relative quantification of CD86, (D), and CD206 after different concentration treatments. (E, G) Flow cytometry analysis of the population of M1/M2-macrophage after different concentration treatments. (F, H) The percentages of M1-macrophage (F4/80 + CD86+) and M2-macrophage (F4/80+ CD206+). (I) Secretion of TNF-a, IL-10, and IL-6 after different concentration treatments. Reproduced with permission 66. Copyright 2024, Elsevier. (C) M@C-HA/ICG and macrophage polarization. (a, b) The ratio of M1- macrophages after being treated with LPS and C-HA/ICG. (c) The generation of ROS, NO, TNF-α, and IL-12 after RAW264.7 cells were treated with different concentrations. (d) The release of LDH from 4T1 cells after treatment with M or M@C-HA/ICG. (e) Fluorescent images of 4T1 cells labeled with calcein-AM/PI after co-culture with M or M@C-HA/ICG. (f) Toxicity of M@C-HA/ICG to 4T1 cells. Reproduced with permission 60. Copyright 2022, Elsevier. (D) *In vivo* macrophage polarization for a pro-inflammatory TME in tumor mice. (A) The mRNA levels of M1 (iNOS and TNF-α) and M2 markers (Arg-1 and IL-10) within the tumor tissues using qRT-PCR. (B) TAM repolarization: representative immunofluorescence images of iNOS (red) and Arg-1 (red) from confocal microscopy. Reproduced with permission 65 copyright 2022, American Chemical Society.
