# Macrophage membrane-functionalized nanotherapeutics for tumor targeted therapy: Erratum

**DOI:** 10.7150/thno.120915

**Published:** 2026-02-21

**Authors:** Mubassir Khan, Razi Ullah, Guixue Wang, Maoquan Chu

**Affiliations:** 1 Key Laboratory of Biorheological Science and Technology of Ministry of Education, College of Bioengineering, Chongqing University, Chongqing, 400044, P.R. China.; 2 Key Laboratory of Biorheological Science and Technology of Ministry of Education, State and Local Joint Engineering Laboratory for Vascular Implants, Bioengineering College of Chongqing University, Jinfeng Laboratory, Chongqing, 400030, P.R. China.; 3 Research Center for Translational Medicine at Shanghai East Hospital, Frontier Science Center for Stem Cell Research, School of Life Sciences and Technology, Tongji University, Shanghai, 200092, P.R. China.

This is a review paper. All the experimental data were cited from the published literatures.

1. The Figure 5 on page 4831 is inappropriate, therefore this Figure 5 should be replaced by a new one, and the corresponding figure caption should also be changed. The new Figure 5 and its caption are as follows.

A phrase “(Figure 5)” should be added at the end of the last sentence of first paragraph on page 4830, that is:

The original sentence: Here we discuss different strategies and methodologies of MΦM-coated nanotherapeutics for cancer immunotherapy.

The revised sentence: Here we discuss different strategies and methodologies of MΦM-coated nanotherapeutics for cancer immunotherapy (Figure 5).

2. A phrase “for multipurpose (Figure 2)” should be added at the end of the last sentence on page 4825:

The original sentence: but they can also evade the immune system.

The revised sentence: but they can also evade the immune system for multipurpose (Figure 2).

The authors are sorry for the Corrigendum.

## Figures and Tables

**Figure 5:**
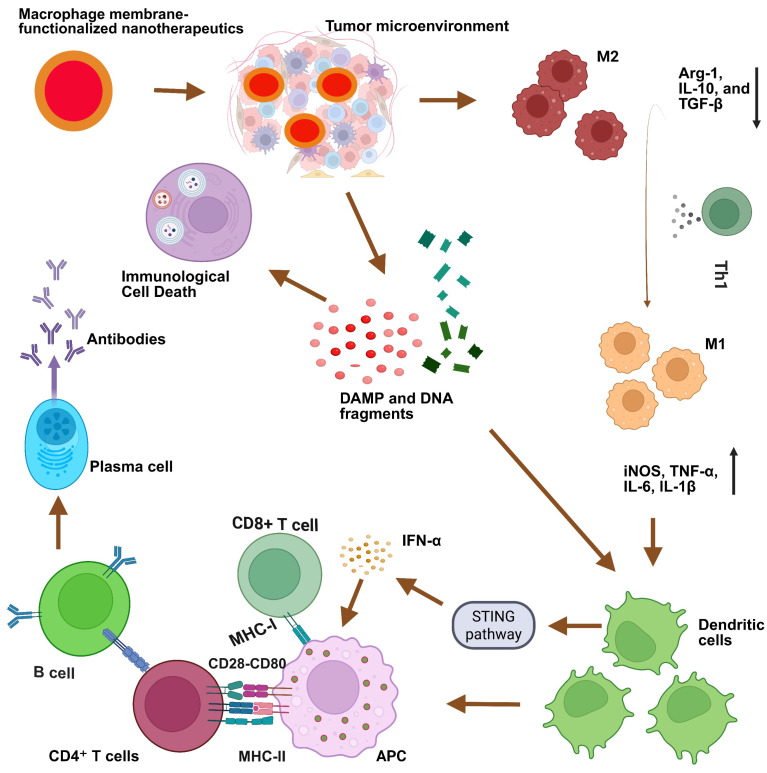
Schematic illustration of MΦM-functionalized nanotherapeutics to modulate tumor immunotherapy

